# The Effect of Temperature over the Growth and Biofilm Formation of the Thermotolerant *Aspergillus flavus*

**DOI:** 10.3390/jof11010053

**Published:** 2025-01-10

**Authors:** José Alejandro Hernández-Benítez, Brenda Nallely Santos-Ocampo, Daniel Genaro Rosas-Ramírez, Luis Antonio Bautista-Hernández, Víctor Manuel Bautista-de Lucio, Néstor Octavio Pérez, Aída Verónica Rodríguez-Tovar

**Affiliations:** 1 Departamento de Microbiología, Escuela Nacional de Ciencias Biológicas, Instituto Politécnico Nacional, Prol. Carpio y Plan de Ayala s/n Col. Casco de Santo Tomás, Alcaldia Miguel Hidalgo, Mexico City C.P. 11340, Mexico; jhernandezb2100@alumno.ipn.mx (J.A.H.-B.); bsantoso1400@alumno.ipn.mx (B.N.S.-O.); 2 Departamento de Química de Biomacromoléculas, Instituto de Química, Universidad Nacional Autónoma de México, Av. Universidad 3000, Circuito Exterior s/n, Ciudad Universitaria, Alcaldía Coyoacán, Mexico City C.P. 04510, Mexico; dgrosas@unam.mx; 3 Unidad de Investigación del Instituto de Oftalmología, Fundación de Asistencia Privada Conde de Valenciana I.A.P., Chimalpopoca 14, Col. Obrera, Alcaldía Cuahutémoc, Mexico City C.P. 06800, Mexico; luis.bautista@institutodeoftalmologia.org (L.A.B.-H.); vbautistal@institutodeoftalmologia.org (V.M.B.-d.L.); 4 Departamento de Investigación y Desarrollo, Probiomed, S.A. de C.V., Cruce de Carreteras Acatzingo-Zumahuacan s/n, Tenancingo C.P. 52400, State of Mexico, Mexico

**Keywords:** *Aspergillus flavus*, biofilm, thermotolerant, extracellular matrix, lipid droplets, antifungal susceptibility

## Abstract

*Aspergillus flavus* is a medically relevant fungus, particularly in tropical regions. Although its aflatoxin production and thermotolerance are well documented, its biofilm-forming ability has received less attention, despite being a key factor in the virulence of *A. flavus* as an opportunistic pathogen, which can significantly impact therapeutic outcomes. To investigate the influence of temperature on the growth and biofilm formation of an *A. flavus* isolate, we compared it on solid media with the reference strain *A. flavus* ATCC 22546 and documented morphological changes during conidial germination. We examined biofilm formation in both strains across different temperatures and evaluated the susceptibility of this *A. flavus* isolate to antifungal agents in both planktonic and biofilm form. Our results showed that the temperature can promote conidiation on solid media. Radial growth was highest at 28 °C, while the conidial count and density were favored at higher temperatures. Moreover, we determined that 37 °C was the optimal temperature for conidial germination and biofilm formation. We described four distinct phases in *A. flavus* biofilm development—initiation (0–12 h), consolidation (12–24 h), maturation (24–48 h), and dispersion (48–72 h)—with the notable presence of conidial heads at 42 °C. Carbohydrates and proteins constitute the primary components of the extracellular matrix. We observed an abundance of lipid droplets within the hyphae of the MMe18 strain biofilm. The mature biofilms demonstrated reduced susceptibility to amphotericin B and itraconazole, requiring higher inhibitory concentrations for both antifungals compared with their planktonic counterparts.

## 1. Introduction

The genus *Aspergillus*, comprising over 200 species of filamentous fungi, is ubiquitous in nature, with members commonly found in air, water, and soil. They can develop as saprobes on decaying organic matter [1]. Nevertheless, species grouped in the *Fumigati*, *Flavi*, *Nigri*, *Terrei*, and *Nidulantes* sections have been described as opportunistic pathogens of humans and animals. Among these, *Aspergillus fumigatus* is the main medically relevant species and the principal etiological agent of invasive pulmonary aspergillosis (IPA) [2]. *Aspergillus flavus* is the second most frequently associated species with IPA, especially in warm-weather countries [3,4]. Moreover, the pathogenic spectrum of *A. flavus* is broader and extends beyond pulmonary aspergillosis, including fungal keratitis [5,6,7], aspergilloma [8,9,10,11], and skin infections [12,13,14]. Furthermore, *A. flavus* is a major source of fungal contamination in grain and corn warehouses, resulting in economic losses by product deterioration and posing a health risk to humans and animals due to its production of mycotoxins, including aflatoxin B1 (AFB1) and aflatoxin B2 (AFB2), with AFB1 being associated with liver and lung cancer [15,16,17,18]. The ability to grow across a broad range of temperatures is one of the virulence factors of the *Aspergillus* genus. This allows them to be found at diverse geographical locations [19] and can favor their capacity to colonize, germinate [20], and develop inside mammalian respiratory tracts, acting as pathogens [21,22]. In fact, the relationship between tropical climatic conditions and the prevalence of infections associated with *A. flavus* has been documented [23,24]. This phenomenon is attributed to the adaptability of the fungus in both decaying organic matter and within the human body, driven by changes in genetic regulation which lead to various morphophysiological adaptations [25], such as the expression of heat shock proteins [26], solute accumulation [27], and cell wall remodeling [28]. Another feature which highlights the adaptive capacity of microorganisms is their ability to form biofilms [29]. Biofilm formation and its role in virulence and resistance to various factors, including antimicrobial therapy and the immune system, have been extensively documented in fungi such as *Candida albicans* [30,31] and *A. fumigatus* [32,33]. Due to its medical importance, significant efforts have been directed toward the characterization of biofilm in these models. However, the rise of opportunistic fungal infections associated with emerging or re-emerging fungi is a serious concern, particularly in the context of climate change, which may drive the adaptation of diverse microorganisms to adverse conditions, such as temperature variations [34,35,36]. This study aimed to investigate the impact of temperature on the development of an *A. flavus* strain isolated from a hospital setting. We characterized its growth on solid media and its biofilm formation capacity at three different temperatures. We also assessed its germination rate and biomass production, as well as the biofilm’s architecture, composition, and response to antifungal agents.

## 2. Materials and Methods

### 2.1. Biological Material and Identification

The *Aspergillus* sp. strain was kindly provided by MSc Jesús Reséndiz Sánchez and was originally isolated postmortem from the lung of a pediatric leukemia patient who did not exhibit pneumonia symptoms at the Hospital Infantil de México “Federico Gómez”. It was grown in Sabouraud dextrose agar (SDA) (DIBICO^TM^, Cuautitlán Izcalli, State of Mexico, Mexico), potato dextrose agar (PDA) (BD Bioxón^TM^, Cuautitlán Izcalli, State of Mexico, Mexico), and Czapek Dox agar (CPK) (Sigma-Aldrich, St. Louis, MO, USA) at 28 °C for 7 days to describe its colonial morphology. Description of the asexual and sexual reproductive structures was performed using a microculture at 28 °C for 7 days. Additionally, where indicated, experiments were conducted with the reference strain *A. flavus* Link var. *flavus* ATCC 22546, an aflatoxin-producing strain originally isolated from moldy corn [37] and kindly provided by Dr. Carlos Cabello Gutiérrez from the Instituto Nacional de Enfermedades Respiratorias (INER) “Ismael Cosio Villegas”. Both strains were assessed for their mycotoxin production potential [38]. The *A. flavus* ATCC 22546 strain was confirmed as a mycotoxin producer, whereas the MMe18 isolate did not produce aflatoxins under the tested conditions.

Molecular identification was performed through genomic DNA extraction following the protocol described by Rodríguez-Tovar et al. [39], followed by PCR amplification of the ITS region and β-tubulin encoding gene. The amplification was conducted using an Axygen^TM^ MaxyGene II thermal cycler (Corning, NY, USA), with a reaction mixture containing 100 ng of a DNA template, 20 pmol of each primer (ITS1 (5′-TCCGTAGGTGAACCTGCGG-3′) and ITS4 (5′-TCCTCCGCTTATTGATATGC-3′) for the ITS region [40] and Bt2a (5′-GGTAACCAAATCGGTGCTGCTTTC-3’) and Bt2b (5’-ACCCTCAGTCTAGTGACCCTTGGC-3′) for the β-tubulin) [41], and 25 μL 2X PCR SuperMix^TM^ (GeneDirex, Taoyuan, Taiwan), for a final volume of 50 μL. The following conditions were used: an initial denaturation step at 95 °C for 10 min, followed by 35 cycles of 1 min at 95 °C, 1 min at 56.7 °C for the ITS and 55 °C for the β-tubulin, and 1 min at 72 °C, with a final extension step at 72 °C for 10 min. The PCR products were sequenced in MacroGen Inc. (Seoul, Republic of Korea). The sequences were edited and aligned using BioEdit (version 7.2.5) and the Clustal Omega server (https://www.ebi.ac.uk/jdispatcher/msa/clustalo, accessed on 25 April 2024), respectively. Subsequently, the sequences were analyzed using BLASTn (https://blast.ncbi.nlm.nih.gov/, accessed on 27 April 2024), and a phylogenetic tree was constructed using the neighbor-joining method in MEGA 11 (version 11.0.8).

### 2.2. Aspergillus flavus Growth at Different Temperatures

The strain was identified as *A. flavus* MMe18. Both the *A. flavus* MMe18 strain and the reference strain *A. flavus* ATCC 22546 were grown on PDA medium at 28 °C for 7 days. Then, the conidia were harvested using 1X phosphate-buffered saline (PBS) (8 g NaCl, 0.2 g KCl, 1.37 g NaH_2_PO_4_, and 0.24 g KH_2_PO_4_) with 0.1% Tween 20 (Hycel de Mexico, Zapopan, Jalisco, Mexico). The conidial suspension was counted using a hemocytometer and adjusted to an inoculum size of 1 × 10^6^ conidia/mL in 1X PBS. The fungal inoculum was spotted onto Petri dishes containing PDA, SDA and CPK and incubated at 28 °C, 37 °C, and 42 °C for 7 days. The colony diameter, total conidia, and conidium-to-colony area ratio were determined at the end of the incubation period in triplicate. For subsequent experiments, these conditions for the fungal inoculum, culture medium, and incubation temperature were used, except where otherwise noted.

### 2.3. Germination of Aspergillus flavus MMe18 Conidia

A conidial suspension was adjusted to 1 × 10^6^ conidia/mL in supplemented RPMI 1640 medium (Gibco^TM^, Thermo Fisher Scientific, Waltham, MA, USA). The adjusted inoculum was seeded into 12 well polystyrene plates (Santa Cruz Biotechnology, Dallas, TX, USA) and incubated at 28 °C, 37 °C, and 42 °C. Fungal development was monitored for 10 h using an inverted microscope (Primovert, Zeiss, Jena, Germany) at a 400× total magnification. The germination percentage was determined by counting both germinated and non-germinated conidia in random fields from three independent replicates for each time point and temperature, as described by Cortesão et al. [42]. Two-way ANOVA and the Holm–Sidak multiple comparisons test were performed using GraphPad Prism (version 9.5.0), with statistical significance is indicated by *, *p* < 0.05.

Metabolic activity during conidial germination was assessed using the MTT assay, as described by Córdova-Alcántara et al. [43]. Fungal inoculum adjusted at 1 × 10^6^ conidia/mL was seeded into 96 well polystyrene plates (Nunc^TM^, Thermo Scientific^TM^, Waltham, MA, USA) and incubated at 28 °C, 37 °C, and 42 °C. After 4 h, 6 h, 8 h, and 10 h, the supernatant was removed, and 100 μL of 0.3% MTT (Invitrogen^TM^, Thermo Fisher Scientific, Waltham, MA, USA) and 100 μL of 1X PBS were added and incubated at 37 °C in the dark for 2 h. Afterward, the supernatant was removed, and 100 μL of dimethyl sulfoxide (DMSO) (Honeywell, Morrison, NJ, USA) and 25 μL of 0.1 M glycine buffer (pH: 10.2) were added and incubated at room temperature for 15 min with mild shaking. Absorbance was measured using a microplate reader (UT-2100C MRC Laboratory Instruments, Holon, Israel) at a wavelength of 450 nm. Differences in absorbance between temperatures were analyzed by two-way ANOVA, followed by the Holm–Sidak multiple comparisons test, using GraphPad Prism (version 9.5.0), with statistical significance indicated by *, *p* < 0.05.

### 2.4. Aspergillus flavus MMe18 In Vitro Biofilm Formation

The conidial suspension was adjusted to 1 × 106 conidia/mL in supplemented RPMI 1640 medium as previously described. The biofilm was established as follows. First, 200 μL of the conidial suspension was added to each well in a 96 well polystyrene plate, resulting in a final conidial density of 2 × 105 conidia per well, and incubated at 28 °C, 37 °C, and 42 °C for 4 h to allow the adherence phase. Then, the supernatant was removed and replaced with fresh RPMI 1640 medium. The biomass was quantified at each temperature over 72 h following the method described by Christensen et al. [44] and modified by Peeters et al. [45] and Ramírez-Granillo et al. [46] as follows. After incubation, the supernatant was removed, and the biofilm was rinsed twice with 200 μL of 1X PBS, fixed with 200 μL of methanol for 15 min, and stained with 200 μL of 0.005% crystal violet (CV) for 20 min, after which it was rinsed with water and allowed to air dry. The dye crystals bound to the biofilm were dissolved using 33% (*v*/*v*) acetic acid, the plates were incubated at room temperature for 15 min, and the absorbance was measured using a microwell reader at a wavelength of 630 nm. Differences in absorbance were analyzed by two-way ANOVA and the Holm–Sidak multiple comparisons test using GraphPad Prism (version 9.5.0). The biofilm formation capacity of the reference strain *A. flavus* ATCC 22546 was studied as well and compared with that of the MMe18 strain.

Additionally, we studied the *A. flavus* MMe18 biofilm morphology through 0.005% CV staining and observed it using an inverted microscope. Then, we determined the biofilm weight by establishing the biofilm in a 6 well polystyrene plate (Ultra Cruz^TM^, Santa Cruz Biotechnology, Dallas, TX, USA) and incubating it at 28 °C, 37 °C, and 42 °C for 48 h. After this period, the supernatant was discarded, and the biomass was rinsed twice with 1X PBS, freeze-dried, and weighed.

### 2.5. Description of the Aspergillus flavus MMe18 Biofilm Architecture

The fungal biofilm was established as described previously in 12 well polystyrene plates and incubated at 28 °C, 37 °C, and 42 °C. Observations were made using scanning electron microscopy (SEM) at 4 h, 8 h, 12 h, 24 h, 48 h, and 72 h. After each incubation period, the supernatant was discarded, and the biofilm was rinsed with 1X PBS and then fixed with 2.5% glutaraldehyde (Sigma-Aldrich, St. Louis, MO, USA) for 2 h, rinsed twice, and post-fixed with 1% osmium tetroxide (Sigma-Aldrich, St. Louis, MO, USA) for 2 h. Following the fixation and post-fixation processes, the samples were rinsed again, dehydrated with increasing concentrations of ethanol, and finally dried to the critical point using 1,1,1,3,3,3-hexamethyldisilazane (Electron Microscopy Sciences, Hatfield, PA, USA).

The samples were coated with ionized gold for 50 s at 5.0 kV and 10 mA and observed using SEM (FEI Quanta 3D FEG Dual Beam, FEI Company, Hillsboro, OR, USA) at the Dual Beam Scanning Electron Microscopy Laboratory, and a JSM-7800F (JEOL Ltd., Tokyo, Japan) at the High-Resolution Scanning Electron Microscopy Laboratory of the Nanosciences, Micro and Nanotechnologies Centre at the National Polytechnic Institute (CNMN-IPN) in Mexico City. The samples were visualized according to scheduled appointments in the CNMN-IPN.

### 2.6. Qualitative Microscopic Detection of the Extracellular Matrix Components

The biofilm of *A. flavus* MMe18 was established as previously described on sterile glass coverslips (Velab^TM^, Mexico City, Mexico) placed at the bottom of a well in a 12 well polystyrene plate and incubated until the maturation phase (48 h) at 28 °C, 37 °C, and 42 °C. After this period, the supernatant was removed, and the biofilm was rinsed twice with 1X PBS. The samples were fixed with 4% paraformaldehyde (Sigma-Aldrich, St. Louis, MO, USA) for 2 h. Afterward, the biofilm was rinsed twice and covered with different mixtures of fluorochromes, namely M1 (1 g/L calcofluor white (CW, blue) (Sigma-Aldrich, St. Louis, MO, USA) + 10x Flamingo^ΤΜ^ (green) (Biorad, Hercules, CA, USA)) to label the chitin and proteins, respectively, and M2 (1 mg/mL concanavalin A (ConA, green) (Sigma-Aldrich, St. Louis, MO, USA) + 100 μg/mL propidium iodide (PI, red)) to label the carbohydrates and extracellular DNA (eDNA), respectively. The presence of lipids in the biofilm was studied through staining with 10 mg/L Nile red (NR, orange) (Sigma-Aldrich, St. Louis, MO, USA). All samples were observed using an epifluorescence microscope (LSM Carl Zeiss, Germany) at the Ocular Microbiology and Proteomics Laboratory of the Ophthalmology Institute “Fundación Conde de Valenciana” in Mexico City. The filters used were CW (355–433 nm); Flamingo^TM^ (512–535 nm); ConA (495–519 nm); PI (543–560 nm); NR (543–560 nm).

Additionally, mature biofilms of *A. flavus* MMe18 and ATCC 22546 were stained with Sudan Black B (Sigma-Aldrich, St. Louis, MO, USA) and examined using brightfield microscopy. After incubation, the supernatant was removed, and the biofilms were washed with 1X PBS. Then, they were stained with 0.5% Sudan black B in 70% ethanol and dried at 50 °C for 20 min. Excess dye was removed with 70% ethanol, followed by counterstaining with 0.5% safranin. The samples were rinsed with distilled water, air-dried, and observed using a brightfield microscope (PrimoStar, Zeiss, Jena, Germany).

To assess whether the biofilm density varied with the temperature, the *A. flavus* MMe18 mature biofilms were stained with calcofluor white and analyzed using an LSM 710 NLO confocal laser scanning microscope (CLSM) (Carl Zeiss, Jena, Germany) at the Multiphoton-Confocal Microscopy Laboratory of the CNMN-IPN in Mexico City. Images were captured using ZEN 2.3 SP1 software (Carl Zeiss, Jena, Germany) and acquired as Z-stacks to generate three-dimensional reconstructions. The excitation laser was set to 405 nm with 2.0% transmittance, and a fluorescence signal was detected within the 41–466 nm range. The three-dimensional images were color-coded, with distinct colors indicating varying depths.

### 2.7. Antifungal Susceptibility Profile of A. flavus MMe18

The minimum inhibitory concentration (MIC) of amphotericin B (AFB) and itraconazole (ITR) against both planktonic and biofilm fungal cells was determined at three temperatures. For planktonic determination, we followed the broth microdilution method for filamentous fungi described in the M38-A document [47] of the Clinical and Laboratory Standards Institute (CLSI). The polystyrene plates were incubated at 28 °C, 37 °C, and 42 °C. Results were determined spectrophotometrically after 48 h of incubation using a microplate reader at a wavelength of 405 nm with agitation. The MICs of both antifungals were determined as the lowest concentration which visibly inhibited fungal growth or resulted in a 50% reduction in the optical density (OD) compared wuth the growth control [47].

For antibiofilm activity, the biofilm was established as previously described in a 96 well plate and incubated at different temperatures. After 24 h of incubation, the supernatant was removed, and the preformed biofilm was exposed to the antifungals. The plates were then incubated until they reached a total of 72 h of incubation. Metabolic activity was measured with the MTT assay.

## 3. Results

### 3.1. Morphological and Molecular Identification

**Figure 1:**
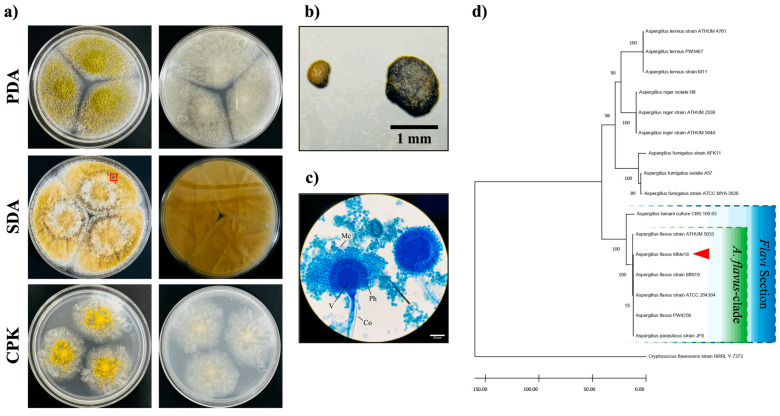
Identification of *Aspergillus flavus* MMe18. (**a**) Macroscopic identification, with front and back views of *Aspergillus flavus* MMe18 grown at 28 °C for 5 days on PDA, SDA, and Czapek agar (CPK). There are downy texture colonies in light green to yellow on the surface and no diffusible pigmentation, (**b**) sclerotia are prominently visible in SDA medium (red box), and (**c**) microscopic identification observed at 1000× magnification, with lactophenol cotton blue stain showing partially rounded conidial heads and uniseriate phialides from which chains of round microconidia develop. (**d**) ITS neighbor-joining phylogenetic tree with a bootstrap value of 1000, with grouped, isolated MMe18 (red arrowhead) within the *A. flavus*-clade in the *Flavi* section. The scale bars indicate 1 mm (**b**) and 20 μm (**c**). Co = conidiophore; V = vesicle; Ph = phialide; Mc = microconidia.

The *Aspergillus* sp. isolate was cultured on different media and incubated at 28 °C for 5 days, as shown in Figure 1a. The colonies exhibited a flat surface and downy texture, with light green to yellow coloration and a white halo at the border. No diffusible pigmentation was observed on the reverse sides of the colonies. Notably, in the SDA medium, the colonies produced a considerable number of dark sclerotia, each larger than 400 μm (Figure 1b). Microscopically, the fungus exhibited conidiophores with partially rounded conidial heads, from which uniseriate phialides and chains of conidia were arranged at an angle close to 360° (Figure 1c). Molecular identification was performed by sequencing and aligning the ITS1–5.8S-ITS2 rDNA fragment with accession number PQ269296. The phylogenetic tree constructed using the neighbor-joining method (Figure 1d) shows that this *Aspergillus flavus* strain (red arrowhead) was grouped within the *A. flavus*-clade (green box), along with other *A. flavus* and *A. parasiticus* isolates. Furthermore, these taxa were situated within the *Flavi* section (blue box), which included *A. tamarii*. This was further supported by constructing an additional phylogenetic tree using a partial sequence of the β-tubulin coding gene (PQ306549) (Figure S1). For subsequent experiments, the *A. flavus* isolate will be referred as *A. flavus* MMe18.

### 3.2. Optimal Growth Conditions for Aspergillus flavus

**Figure 2:**
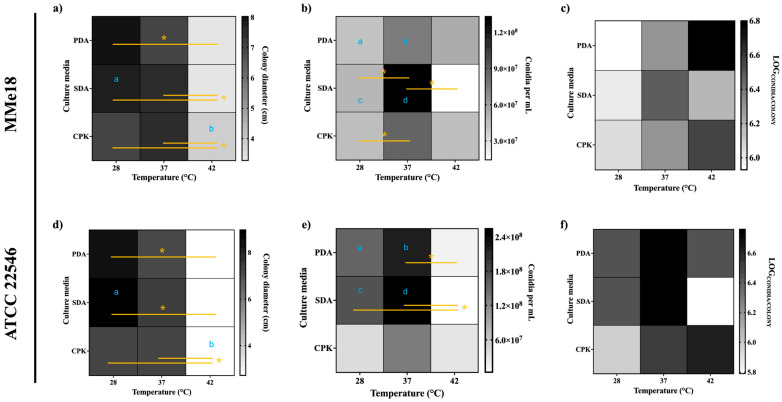
*Aspergillus flavus* growth under different conditions. Fungal development of both *A. flavus* MMe18 and *A. flavus* ATCC 22546 is depicted in terms of colony diameter (**a**,**d**), total conidia (**b**,**e**), and the logarithm of the conidia/colony area ratio (**c**,**f**) after 7 days of incubation across different media and temperatures. Darker boxes indicate higher values. The optimal temperature for radial growth in both *A. flavus* strains was 28 °C, and it decreased as the temperature rose. In contrast, conidiation was enhanced at 37 °C and 42 °C, as shown by the conidiation per colony area calculation. Significant differences were assessed using independent two-way ANOVA analyses followed by a Holm–Sidak multiple comparisons test. These are indicated in yellow, *, *p* < 0.05 for comparisons between temperatures within the same strain and blue, letters a, b, c, and d for comparisons between different strains.

The development of *A. flavus* MMe18 in different culture media and incubation temperatures was quantified by measuring the colony diameter (Figure 2a), total conidia count (Figure 2b), and the conidia-to-colony area ratio (Figure 2c) after 7 days of incubation. These values were compared with those of the reference strain *A. flavus* ATCC 22546 (Figure 2d–f). The optimal temperature for radial growth in both strains was 28 °C, followed by 37 °C. Although the colony diameter significantly decreased across all media at 42 °C, both strains continued to grow, with conidial counts exceeding 10^7^ conidia/mL. The lowest conidial counts were observed in the Czapek medium, with no significant differences among temperatures within each isolate only in this medium. Notably, in rich media (PDA and SDA), the conidial counts were significantly higher in the reference strain compared with the MMe18 isolate at 28 and 37 °C but not at 42 °C. Regardless of the culture medium, the conidial density increased with the temperature. These findings suggest that the conidiation rate may not directly correlate with radial growth but is influenced by thermal stress at temperatures above the optimal range and by the culture medium. Overall, these results confirm that thermotolerance is a characteristic of the *A. flavus* species. Additionally, we visually compared the growth of these fungal strains on PDA medium at three temperatures with two other *A. flavus* isolates: *A. flavus* PHA, obtained from a *Plodia interpunctella* pupa, and *A. flavus* MT, an isolate from an internal collection (Figure S2).

### 3.3. Physiological Temperature Accelerates Conidial Germination of Aspergillus flavus

**Figure 3:**
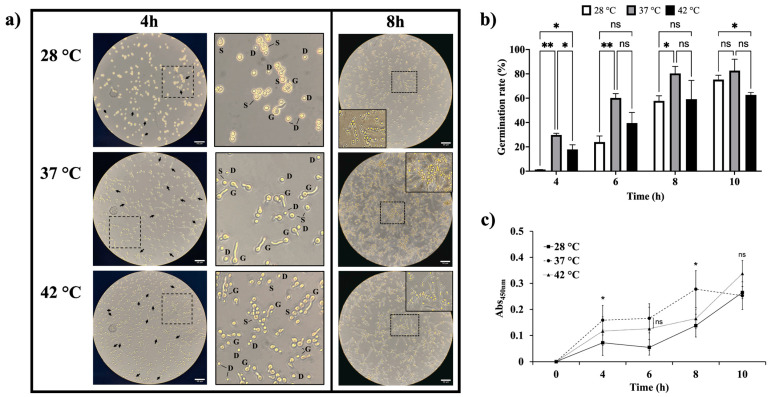
Temperature effect on *A. flavus* MMe18 conidia germination. The inoculum was adjusted to 1 × 10^6^ conidia/mL in RPMI 1640 medium and seeded onto polystyrene plates, followed by incubation at 28 °C, 37 °C, and 42 °C. (**a**) Conidial development observed at 400× total magnification, black arrows indicate germinating conidia observed in each field shown. Solid-line squares represent higher magnification areas within the dotted-line squares, highlighting the crucial role of temperature in breaking dormancy and triggering morphological changes, such as the swelling (S) of dormant conidia (D), leading to germination (G). (**b**) Germination percentage and (**c**) metabolic activity of conidia during the first 10 h of incubation, emphasizing that increasing temperatures stimulate conidial development. Significant differences were determined via two-way ANOVA and a Holm–Sidak multiple comparisons test in both (**b**,**c**) panels as indicated (*, *p* < 0.05; **, *p* < 0.01; ns, not significant). The scale bars indicate 50 μm (**a**).

We noted that the temperature affected both the colony size and the conidiation rate of *A. flavus*. To understand how temperature affects conidial germination, we monitored their development over 10 h in liquid medium at three different temperatures. In Figure 3a, we show that morphological changes associated with germination were present at both 37 °C and 42 °C within 4 h of incubation, being more evident at 37 °C. We observed isotropic growth in the conidia, which appeared swollen (S), and some had already germinated (G). In contrast, at 28 °C, no germinated conidia were observed, and these morphological changes became evident only after 6 h, with many conidia remaining in a dormant state (D). Additionally, hyphal elongation and intertwining occurred sooner at 37 °C and 42 °C compared with 28 °C. After 8 h of incubation at 37 °C, clusters of conidia were already forming microcolonies with emerging hyphae. At 42 °C, hyphae had begun to intertwine, whereas at 28 °C, the elongation process had only just begun. Germination was also assessed at inoculum sizes of 1 × 10^5^ and 1 × 10^7^ conidia/mL (Figure S3). This was corroborated by determining the germination percentage (Figure 3b). During the first 8 h of incubation, the germination percentage increased with rising temperatures, reaching significantly higher levels at 37 °C than at 28 °C. Although incubation at 37 °C favors the germination process, increasing the temperature to 42 °C reduces the germination rate since after 8 h, there was no significant difference in the germination rates between 28 °C and 42 °C. Additionally, the metabolic activity during the germination process was evaluated using an MTT assay. As shown in Figure 3c, the highest absorbance value was observed at 37 °C, suggesting that dormancy breaking and germination occur rapidly at this temperature. Meanwhile, the metabolic activity of the conidia incubated at 28 °C remained low, confirming that the germination process is slower at this temperature.

### 3.4. Aspergillus flavus In Vitro Biofilm Is Favored by Temperature

**Figure 4:**
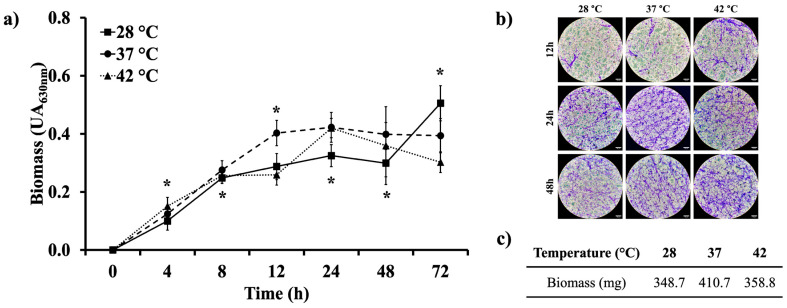
*Aspergillus flavus* MMe18 in vitro biofilm. Fungal biofilms were established and incubated at different temperatures for 72 h. (**a**) Biomass was quantified using crystal violet staining across three independent experiments. The values in the graph represent the mean ± SD. Differences between means were assessed using two-way ANOVA followed by a Holm–Sidak multiple comparisons test, with significant differences indicated by *, *p* < 0.05. Regardless of the development temperature, *A. flavus* MMe18 biofilm kinetics exhibited a similar pattern with four distinct phases: initiation (0–12 h), consolidation (12–24 h), maturation (24–48 h), and dispersion (48–72 h). However, a notable increase was observed at 28 °C after 48 h. (**b**) At 12, 24, and 48 h, the biofilm was stained with crystal violet and observed using brightfield microscopy at 400× total magnification. (**c**) The biomass of the mature biofilm (48 h) was freeze-dried and weighted, confirming that 37 °C was the optimal temperature for biofilm development and biomass production. The scale bars indicate 50 μm (**b**).

Biofilm formation is an important virulence factor of opportunistic fungi. Thus, we assessed the ability of *A. flavus* MMe18 to form biofilm and studied how the temperature influences this process. Figure 4a depicts biofilm formation at different temperatures: 28 °C (squares), 37 °C (circles), and 42 °C (triangles). In all cases, a microbial growth pattern was observed, with continuous growth from 0 to 12 h of incubation, after which it began to slow down, and it peaked at 24 h regardless of the temperature. After 24 h, growth decreased at 37 °C and 42 °C, while at 28 °C, the biomass significantly increased from 48 to 72 h, reaching the highest recorded biomass, which may be linked to conditions which promote biofilm stabilization under these conditions. Biofilms were stained with crystal violet (CV) and examined microscopically. As shown in Figure 4b, the biofilm morphology was similar across all temperatures after 12 h, featuring hyphal networks and clusters. Between 24 and 48 h, these networks became denser, and hyphal anastomosis was especially evident at 37 °C. The biofilm growth kinetics and fungal biomass staining suggest that 37 °C is the optimal temperature for *A. flavus* MMe18 biofilm formation, which was confirmed by the biomass weight measurements shown in Figure 4c, where the highest biomass production was observed at 37 °C and the lowest was observed at 28 °C.

**Figure 5:**
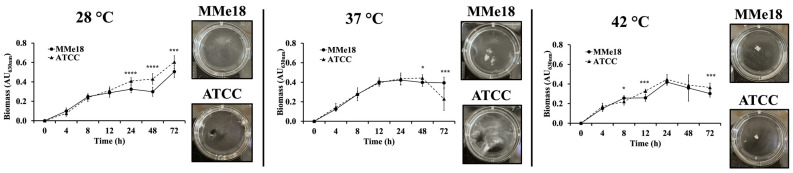
Biofilm formation by *Aspergillus flavus* MMe18 and ATCC 22,546 at various temperatures. Biofilms of both fungal strains were incubated at different temperatures for 72 h, and the biomass was quantified as previously described. Values in the graph represent the mean (*n* = 16) ± SD. Differences between means were analyzed using two-way ANOVA followed by a Holm–Sidak multiple comparisons test, with significant differences indicated by *, *p* < 0.05; ***, 0.0003; ****, <0.0001. Both *A. flavus* MMe18 and *A. flavus* ATCC 22546 showed similar trends. However, *A. flavus* ATCC 22546 displayed a notable reduction in biofilm biomass at 37 °C after 72 h, whereas our strain (MMe18) maintained significantly higher values. This biomass reduction at 37 °C coincides with decreased biofilm stability, as illustrated in the top-down photograph shown in the side box.

We also compared the biofilm formation capacity of *A. flavus* MMe18 with the reference strain *A. flavus* ATCC 22546. Figure 5 presents the biofilm growth curves for both strains, highlighting minimal differences in their growth patterns. At 28 °C, both strains exhibited a substantial increase in biomass after 48 h. However, at 37 °C and 42 °C, the biomass peaked between 24 and 48 h before starting to decline. Notably, after 48 h, the reference strain exhibited a marked decrease in biomass, whereas the MMe18 strain remained in a stationary phase. We attribute this decrease to the cohesive morphology of the *A. flavus* ATCC 22546 strain biofilm at 37 °C, as observed in the side box, which includes a top-down photograph of the biofilm. While it produced a substantial amount of biomass, its weak adherence to the surface makes it prone to detachment during pipette manipulation, as shown in the boxes on the right.

### 3.5. Architecture of the Aspergillus flavus Biofilm

**Figure 6:**
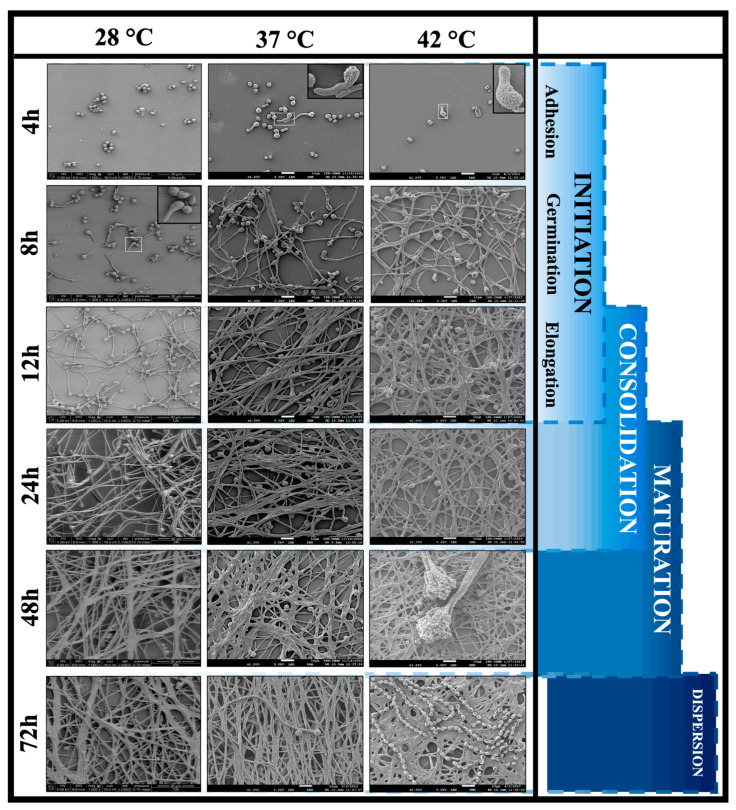
Architecture of *Aspergillus flavus* MMe18 in vitro biofilm. *A. flavus* biofilm was established and incubated at different temperatures. Its development was monitored for 72 h using scanning electron microscopy. Initiation (0–12 h) involves conidial adhesion to the surface during the first 4 h. Along with this phenomenon, conidial germination took place at 37 °C and 42 °C but not at 28 °C, and it is evident that breaking dormancy triggered morphological and structural changes on the conidial surface. After 12 h, hyphal elongation was extensive, and they had crisscrossed forming networks. Consolidation (12–24 h) is where hyphal networks grow and form a three-dimensional structure which increases its thickness and density. Some hyphae were joined by anastomosis, and an extracellular matrix was secreted as well. At this point, the topology of the biofilm was indistinguishable regardless of the temperature of development. When the biofilm matured (48 h), its topology was well defined, featuring water channels and a thick multilayer of interconnected hyphal networks. Conidial heads were observed only at 42 °C. After 72 h, the biofilm lost thickness as it entered into the fungal dispersion phase (>72 h), marking the beginning of the cycle. The black-lined squares indicate higher magnification areas within the white-lined squares. All images were observed at a 1000× total magnification. The samples were visualized according to schedule appointments in the CNMN-IPN.

Scanning electron microscopy (SEM) was employed to study the biofilm formation process of *A. flavus* MMe18 in greater detail, with a focus on understanding how the temperature influences its architecture and topology. Figure 6 shows slight changes in the architecture of the biofilm when it developed at different temperatures. It has been reported that conidial adhesion to the substrate occurs within the first 4 h of incubation. We observed that higher temperatures accelerated germination, as detected at both 37 °C and 42 °C, which corroborates the results shown in Figure 3a. The high-magnification images in the white boxes reveal the surface remodeling in conidia as they begin germination, shedding their hydrophobic protein layer. After 8 h at 28 °C, some conidia had germinated, whereas at 37 °C and 42 °C, elongated, interwoven hyphae were observed, a process which occurred later at 28 °C. By 12 h, multilayered hyphal networks with evident anastomosis had formed at 37 °C and 42 °C, while at 28 °C, hyphal elongation had just begun. According to Figure 4a, the *A. flavus* biofilm reached the maturation phase between 24 and 48 h, as confirmed by its topological characteristics. After 24 h, a multilayered biofilm was observed at all temperatures, with no significant differences in topology, although pronounced hyphal anastomosis was evident at 37 °C and 42 °C. At 48 h, this morphology persisted, along with the presence of water channels and minimal exopolymeric substance production. At 42 °C, conidial heads and abundant conidia were observed, which may indicate the onset of the dispersion phase, occurring after 72 h.

### 3.6. Microscopic Detection of the Extracellular Matrix Components in the Aspergillus flavus MMe18 Mature Biofilm

**Figure 7:**
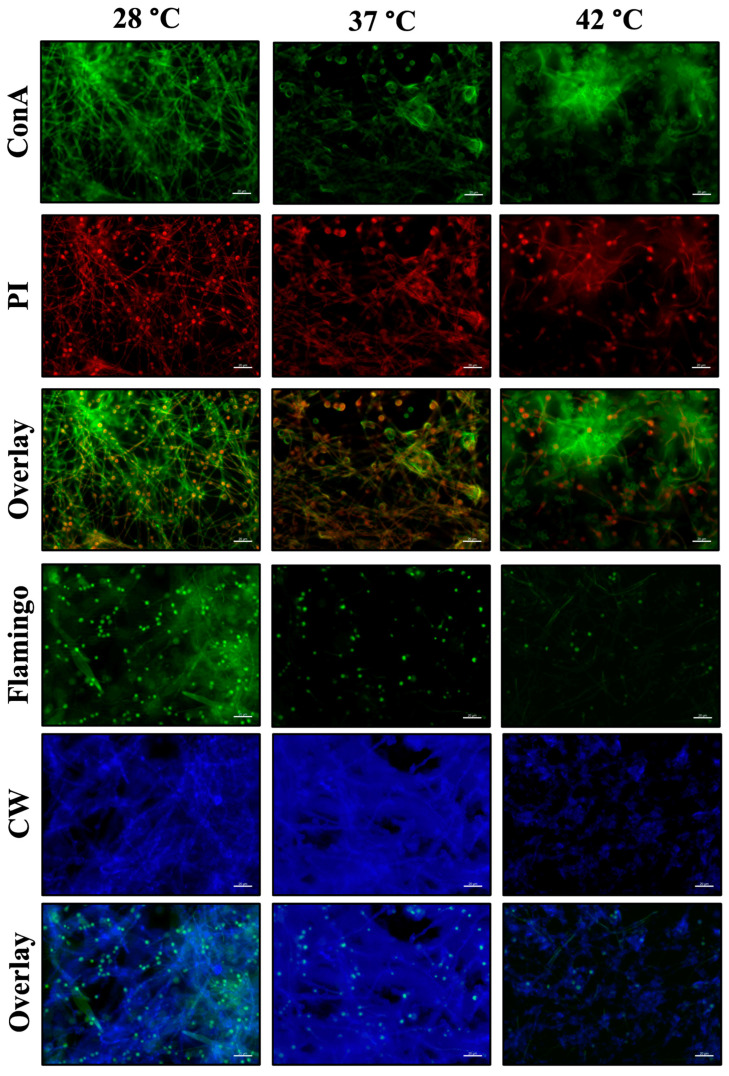
Qualitative detection of biomolecules in the in vitro biofilm of *Aspergillus flavus* MMe18. The mature biofilm was stained to visualize the presence of biomolecules and observed using epifluorescence microscopy at a 400× total magnification. (**upper panel**) The fungal biofilm was stained with concanavalin A (green), which binds to glucosyl and mannosyl residues, and propidium iodide (red), which intercalates into DNA. The red stain was observed within hyphae and conidia, and its presence in the extracellular space indicates that the ECM contained eDNA. The green stain can primarily be seen on conidial and hyphal surfaces composed of glucans and mannans, but it was also present in the surrounding space, suggesting the presence of these molecules in the ECM. (**lower panel**) The biofilm was stained with Flamingo™ (green) to detect proteins and calcofluor white (blue) to label chitin. The blue stain was abundant due to the presence of chitin in both the fungal cells and the ECM. Meanwhile, the green stain was predominantly localized within conidia and, to a lesser extent, inside hyphae and the intercellular space. The scale bars indicate 20 μm. ConA = concanavalin A; PI = propidium iodide; CW = calcofluor white.

We studied the mature biofilm of *A. flavus* MMe18 and compared its biochemical composition at different temperatures using epifluorescence microscopy with two fluorochromes mixtures: M1 (calcofluor white + Flamingo^TM^) and M2 (concanavalin A + propidium iodide). The upper panel of Figure 7 shows the labeling of carbohydrates (green) and eDNA (red) using Con A and PI, respectively. Green labeling with concanavalin A is observed on the hyphal and conidial surfaces, as well as in the extracellular matrix (ECM), where the fungal cells are embedded. Red labeling with PI was detected within the conidia, some hyphae, and the intercellular space, likely due to DNA release during the anastomosis process. The co-localization of the two markers suggests the presence of both biomolecules in the ECM. No significant changes in their relative abundance were detected across the three temperatures evaluated. In the lower section, biofilms labeled with Flaming^TM^ (green) and calcofluor white (blue) are shown, and we observed a homogeneous proportion of protein and chitin, regardless of the incubation temperature. A green label was observed over the hyphae, primarily on the conidia, likely due to the hydrophobic protein layer present on the conidial surface. As previously mentioned, this layer was lost during conidial germination. Co-localization of both fluorochromes was detected in the ECM, particularly in the biofilm developed at 37 °C, where there was an abundant presence of carbohydrates.

**Figure 8:**
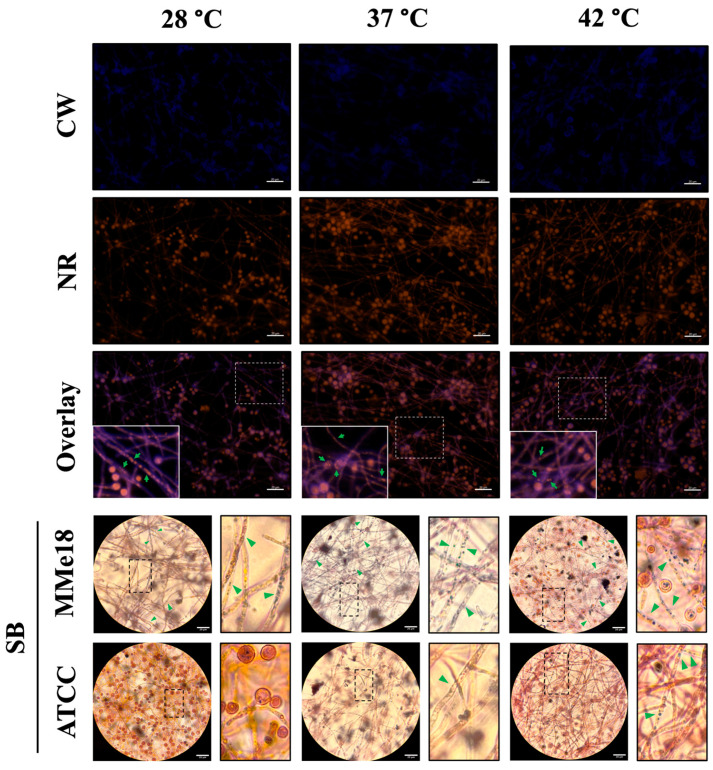
Lipid droplets in the hyphae of *A. flavus* in vitro biofilm. The mature biofilm was stained with calcofluor white (blue) to visualize cell walls and with Nile red (orange) to detect lipids. Samples were observed using epifluorescence microscopy at a 400× total magnification (**upper panel**). Additionally, both strains (MMe18 and ATCC 22546) were stained using Sudan black B staining and observed under brightfield microscopy at a 1000× total magnification (**lower panel**). Lipid staining was observed to a lesser extent in the extracellular matrix, suggesting the minimal structural presence of lipids in the extracellular matrix of the *A. flavus* biofilm, with lipids primarily localized within conidia and along hyphae as droplets, highlighted by green arrows. Notably, lipid droplets (LDs) were predominantly present in the MMe18 strain but not in the reference strain ATCC 22546. Solid-lined squares represent higher magnification areas within the dotted-line squares. The scale bars indicate 20 μm (**upper panel**) and 20 μm (**lower panel**). CW = calcofluor white; NR = Nile red; SB = Sudan black.

Additionally, dual carbohydrate and lipid labeling was performed in the MMe18 strain mature biofilm using CW and Nile red (NR). The upper panel of Figure 8 illustrates the labeling of chitin (blue) in the ECM and on the surfaces of the hyphae and conidia, contrasted with the lipid labeling by Nile red (orange) within the fungal cells, highlighting the presence of lipid inclusions (green arrows). While lipid labeling was prominent inside the hyphae and conidia, its presence was practically absent in the intercellular space, except for some specific accumulations. Thus, we infer that its role in the *A. flavus* biofilm is not merely as a structural component. Furthermore, Sudan black staining was employed to detect the presence of lipid droplets (LDs) (lower panel). When visually comparing the LDs between the two strains, we observed a significantly higher abundance of these structures in the MMe18 strain across all tested temperatures, whereas the ATCC 22546 strain exhibited a lower proportion of LDs under similar conditions.

**Figure 9:**
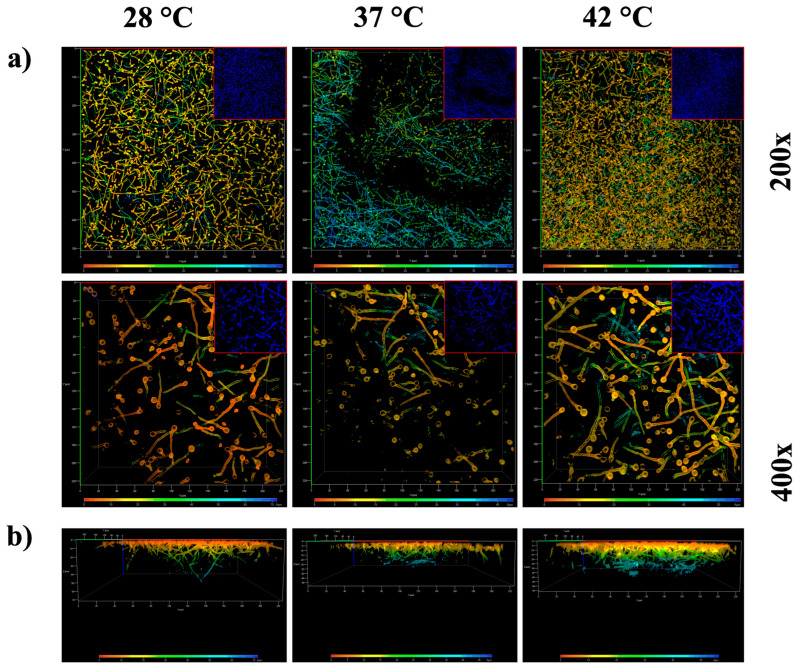
Study of *A. flavus* MMe18 biofilm cell density and thickness. The mature biofilm was stained with calcofluor white and observed using confocal microscopy. (**a**) Three-dimensional views of the mature biofilm observed at 200× and 400× total magnification. The color scale represents depth, with blue tones indicating the greatest depths. It is evident that the hyphal density increased with higher temperatures. The red boxes in the top right correspond to the original, unprocessed micrographs of the biofilm labeled with calcofluor white. (**b**) Z-stack reconstruction, showing that the cell density limits laser penetration. As the depth increased, the cell density decreased, indicating a proportional relationship between temperature and cell density. At 37 °C, the laser penetration power was the lowest.

To study the effect of temperature on the density of the mature biofilm, we used confocal laser scanning microscopy (CLSM). Figure 9a presents the three-dimensional arrangement of the biofilm. When observed at a lower magnification, the cell density increased with the temperature, peaking at 42 °C, followed by growth at 37 °C, and it was the least dense at 28 °C. This is confirmed in the lower panel (Figure 9b). In the Z section of the mature biofilm, it is noted that at 28 °C, the laser penetration was the deepest, with a signal reaching up to 50 μm in depth, whereas at 42 °C, it did not exceed 40 μm, and at 37 °C, it did not exceed 30 μm in depth. This indicates that the cell density was highest at 37 °C, as inferred from the higher resistance to laser penetration by the biofilm.

### 3.7. Antifungal Susceptibility Profile of A. flavus MMe18 as Planktonic Cells or as Biofilm

To determine the in vitro antifungal susceptibility profile of the *A. flavus* MMe18 strain, we assessed its susceptibility to two commonly used antifungals in the treatment of aspergillosis: amphotericin B (AMB) and itraconazole (ITR). We also examined whether this profile varied with temperature changes or during biofilm formation. Table 1 shows the minimum inhibitory concentration (MIC) values of both antifungals at different temperatures. The inhibitory concentration at 28 °C for amphotericin B was determined to be 2 μg/mL, while at 37 °C, it was 4 μg/mL, and at 42 °C, it was 2 μg/mL again. Regarding the itraconazole, the inhibitory concentration under the same conditions was 0.0625 μg/mL at 28 °C and 37 °C and 0.0312 μg/mL at 42 °C. Furthermore, we assessed the antibiofilm activity of both antifungals and observed a reduction in their efficacy against 12 hour-old preformed biofilms (Table S2). When evaluated against mature biofilms (24 h-old preformed), the antifungal activity was further reduced. Itraconazole exhibited MIC values exceeding 16 μg/mL at all tested temperatures, whereas amphotericin B displayed reduced but more variable activity, with an MIC of 8 μg/mL at 42 °C and higher values (16 μg/mL) at both 28 °C and 37 °C.

## 4. Discussion

The *Aspergillus* genus comprises ubiquitous fungi, whose medical importance lies in their opportunistic behavior [48]. *A. flavus* is the second most clinically relevant species after *A. fumigatus* [5,7,23], and it is even more prevalent in tropical and subtropical regions with arid climates [8]. Here, we assessed the optimal conditions for the growth and biofilm formation of *A. flavus*, focusing on how temperature affects its development and response to stressors. The *Aspergillus flavus* isolate, identified as MMe18, was sourced from a necropsy at the “Federico Gómez” Children’s Hospital of Mexico. We observed morphological characteristics consistent with the species *A. flavus*, including the presence of numerous brown sclerotia. Accordingly, this isolate was classified within the L morphotype (sclerotia > 400 μm) [23,49]. Ohkura et al. [50] suggested that the L morphotype is adapted for aerial dispersal and nutrient-limited environments with reduced microbial competition. Molecular identification confirmed the identity of this isolate within the *A. flavus*-clade, which comprises four lineages, primarily *A. flavus* and *A. parasiticus*. This clade is further grouped within the *Flavi* section, where *A. tamarii* is also observed. The *A. flavus*- and *A. tamarii*-clades share a close phylogenetic relationship, and along with the *A. bertholletius*- and *A. nomius*-clades, they form a distinct lineage within the *Flavi* section [51]. When comparing the development of this strain to *A. flavus* ATCC 22546, we noted that radial growth in both strains was inversely proportional to the temperature, while the conidial density increased with the temperature, with *A. flavus* ATCC 22546 showing higher conidial counts. This suggests that conidiation may serve as a dispersion mechanism, promoting fungal survival under thermal stress. Fabri et al. [52] suggested that sphingolipids have a key role in membrane reorganization and signal transduction in response to thermal stress, driven by increased fatty acids and ergosterol, enabling adaptation to elevated temperatures [53]. Here, we observed two key factors which shape the success of opportunistic fungi: microbial burden and thermotolerance. We highlight the capacity of this fungus to proliferate at the human physiological temperature and higher (42 °C), which may provide an adaptive advantage against body defense mechanisms like fever, which enhances the activity of immune cells and molecules, disrupts pathogen integrity, and inhibits their growth through thermal stress, damaging proteins, lipids, and nucleic acids [54]. The ability of microorganisms to grow at body temperature represents a significant health risk, particularly for vulnerable hosts, such as hemato-oncological patients and those with prolonged neutropenia [55,56]. Regarding microbial burden, the size of *Aspergillus* spp. conidia enables them to bypass mucociliary clearance and reach the lower airways, making them successful opportunistic pathogens wjocj pose significant risks even at low fungal burdens [3,19,57]. In a classic study, Ford and Friedman [58] demonstrated an association between inoculum size and lethality in multiple *Aspergillus* species. At an inoculum size of 1 × 10^6^ conidia/mL, *A. flavus* caused 100% lethality in a murine model. Although immunosuppression exacerbated the disease, it was not required for infection establishment. Usman et al. [59] showed that a fungal burden of 1 × 10^4^ conidia/mL of *A. flavus* caused 80% lethality in *C. elegans* and *G. melonella,* while 0.75–1 × 10^8^ conidia/mL was sufficient for paranasal infection in immunocompetent rabbits [60]. Thriving at 37 °C is common in pathogenic microorganisms [21], suggesting a correlation between growth and germination at this temperature and the pathogenicity of *Aspergillus* species [61] as well as their adaptation to hostile conditions [62]. We found that 37 °C was the optimal temperature for *A. flavus* MMe18 conidial germination, with the highest germination percentage and metabolic activity, followed by conidia at 42 °C. The conidia at 28 °C showed delayed germination, taking up to 6 h. Jia et al. [63] reported complete conidial germination of *A. flavus* 12 h at 30 °C. Although we observed accelerated germination at 37 °C, no significant differences were observed after 10 h among the three temperatures, as previously described [64,65]. Kumar et al. [66] reported that *A. flavus* conidia germinate 6–12 h post-inoculation at 26 °C in an in vivo model of fungal infection, consistent with our in vitro observations. Notably, after 8 h of incubation at 37 °C, the germinated conidia formed microcolonies and the basis for hyphal networks, which were more pronounced with an inoculum of 1 × 10^5^ conidia/mL (Figure S3). Fontaine et al. [67] suggested that β-1,3-glucan, which becomes exposed during germination, promotes conidial aggregation. Morelli et al. [68] attributed this to thigmotropism, a well-studied phenomenon in phytopathogenic fungi which was already documented in *A. fumigatus* [69]. Furthermore, we observed inhibition of germination when an inoculum of 1 × 10^7^ conidia/mL was assessed (Figure S3). This phenomenon has been attributed to the release of auto-inhibitors at high cell densities [70]. Conidial germination involves morphological changes leading to the emergence of the germ tube, which implies the activation of metabolic pathways responsible for cell wall remodeling [71]. Under favorable conditions, conidia can germinate, adhere to surfaces, and form microbial consortiums known as biofilms. In nature, biofilm growth represents a survival mechanism, conferring protection against environmental stressors, including those within the human body. Biofilms have been linked to chronic and recalcitrant infections, which are often associated with lower therapeutic success rates [72,73]. To assess the biofilm-forming capacity of *A. flavus* MMe18 and determine the optimal conditions, its development was monitored over 72 h. The curve exhibited continuous growth from 0 to 12 h, with significantly higher biomass quantification at 37 °C. A stationary phase occurred between 12 and 48 h, with peak biomass at 24 h across all temperatures. However, at 28 °C, the growth rate significantly increased after 48 h, possibly due to nutrient availability and favorable conditions which promote prolonged biofilm persistence. Morelli et al. [68] described three basic phases of *A. fumigatus* biofilm development: initiation (0–12 h), immature (12–24 h), and mature (>24 h). Based on these observations, we propose the following phases for *A. flavus* biofilm development. (1) Initiation phase (0–12 h): This includes adhesion, germination and the onset of filamentation, with a continuous increase in biomass. (2) Biofilm consolidation phase (12–24 h): During this phase, biofilm growth stabilizes, and the biomass increases slowly. Active hyphal development and the formation of multilayered networks occur. (3) Maturation phase (24–48 h): The growth rate slows, and the biofilm’s morphology becomes well structured and defined. The biomass reaches its peak. (4) Dispersion phase (48–72 h): This phase is marked by a decrease in biomass and the initiation of conidial dispersion. It was confirmed by studying the biofilm’s morphology. Additionally, the dry weight of the mature biofilm showed the highest biomass at 37 °C, followed by 42 °C and the lowest at 28 °C. González-Ramírez et al. [69] compared biofilm formation by clinical and environmental *A. fumigatus* isolates incubated at 28 and 37 °C, finding that both formed mature biofilms within 24 h, with development favored at 28 °C. They attributed the reduced growth at higher temperatures to thermal stress, regardless of the isolate source. When comparing the biofilm formation of the *A. flavus* MMe18 isolate to that of *A. flavus* ATCC 22546, which was originally isolated from infected corn, no significant differences in the initial development time or growth rate were observed. Notably, after 48 h at 37 °C, the MMe18 isolate maintained stable biomass, whereas the ATCC 22546 strain exhibited a marked decrease. We attribute this to the cohesive morphology of the ATCC 22546 strain biofilm which, although generating substantial biomass, did not adhere strongly to the surface, making it prone to detachment during manipulation. Obana et al. [74] referred to the “pellicle biofilm” formed by *C. perfringens* at 25 °C, which appears thick and viscous but does not adhere strongly to the surface, in contrast to the “adhered biofilm” observed at 37 °C. They concluded that temperature influences biofilm morphology though transcriptional regulation activated at an optimal temperature. These results suggest that at 37 °C, the MMe18 isolate’s biofilm can persist for over 72 h, while the ATCC 22546 strain enters the dispersion phase [75,76]. This may be attributed to adaptive mechanisms in *A. flavus* MMe18 which enable it to thrive at higher temperatures, potentially aiding its establishment in the human body, particularly in hospital environments [21]. It is important to note, however, that thermotolerance appears to be a species-wide characteristic rather than strain-specific. This was demonstrated by assessing the growth of other environmental and reference isolates of *Aspergillus flavus* (Figure S2). Biofilm formation has been widely studied in the *Aspergillus* genus, especially in *A. fumigatus*, due to its clinical significance [77]. Although *A. flavus* biofilm has been studied [78,79,80], no studies to date have provided a comprehensive step-by-step characterization of this process. To our knowledge, this study is the first attempt to characterize the in vitro biofilm of *A. flavus* using an integrative approach. We used scanning electron microscopy (SEM) to provide a detailed description of the biofilm architecture. After 4 h of incubation, adhesion and germination were observed as key events for colonization and biofilm formation. As previously shown, germination occurred earlier at 37 and 42 °C, followed by 28 °C. A higher magnification revealed surface differences between the germinated conidia and germ tubes, likely due to variations in cell wall composition since during germination, the hydrophobic layer is lost, exposing the hyphal cell wall [67,81,82,83,84,85]. It has been shown that cell wall composition and conidial topography are critical factors for adhesion, as they mediate hydrophobic and electrostatic interactions with the surface [83]. Villena and Gutiérrez-Correa [86] reported that the rough conidial surface of *A. niger* facilitates initial fungal-substrate contact. After 8 h, primary hyphal networks formed at 37 and 42 °C. By 12 h, multilayered networks with interhyphal channels marked the onset of biofilm consolidation, at which point susceptibility to stressors decreased [68,87]. At 28 °C, the hyphal monolayer was barely evident. Between 24 and 48 h, minimal topographical differences across the temperatures were observed, although the biofilms exhibited well-defined characteristics. Multilayering intensified, hyphal anastomosis was extensive 37 and 42 °C, and interhyphal channels were more evident, along with EPS production, indicating a mature biofilm. In *A. oryzae*, accelerated mycelial growth, hyphal anastomosis, and tolerance to thermal stress have been linked to the repression of inositol-phosphorylceramide synthase (AoAur1) [88]. At 42 °C, the presence of conidial heads was notable, as observed by Chatterjee and Das [75] during the biofilm dispersion phase in *A. niger*. This likely reflects reduced biofilm formation under nutrient-rich and thermal stress conditions, suggesting that the fungus relies on conidiation and subsequent propagation as a survival strategy. Wu et al. [85] suggested that nutrient limitation triggers a hyphal transition from vegetative growth to reproductive structures, like conidiophores and conidia. Adams et al. [89] noted that hyphae remain vegetative indefinitely unless external stimuli, like air exposure for about 18 h, induce differentiation into reproductive structures. In this context, O2 gradients during biofilm stratification are known to influence morphology and maturation in *A. fumigatus* biofilms. This could explain our observation of conidial heads in the surface layer of mature biofilms at 42 °C [32,90]. Moreover, genes involved in conidiogenesis have been observed to play roles in stress response signaling pathways [91,92]. After 72 h at 42 °C, the biofilms exhibited reduced turgor and thickness, indicating entry to senescence and the dispersion phase. Conversely, at 37 °C, biofilm stratification increased, and germinating conidia were observed, consistent with CV biomass quantification, which showed a stationary phase at 72 h. Thus, we hypothesized that the dispersion phase could extend up to 96 h at 37 °C, as observed in *A. terreus* biofilm by Rayón-López et al. [93]. Studying biofilm formation in *Aspergillus* spp. requires accounting for developmental heterogeneity and the influence of environmental and nutritional factors. *A. fumigatus*, the most clinically relevant species, develops mature biofilms within 24 h, as reported by Mowat et al. [94] and corroborated by our group [46,69]. This likely contributes to its high virulence and prevalence. Other species exhibit longer maturation periods; *A. nidulans*, requires 72 h [76], *A. terreus* may take up to 96 h; and *A. niger* shows variability, maturing as early as 36 h [95] or extending to 96 h, depending on the conditions [86]. This study is the first to detail the in vitro kinetics of *A. flavus* biofilm formation, with maturation occurring between 24 and 48 h and transition into the dispersal phase occurring by 72 h. In this context, the extracellular matrix (ECM) is crucial for biofilm resistance, stability, and long-term persistence, making it a significant area of interest [96,97], comprising carbohydrates and proteins mainly as well as lipids and nucleic acids. Although temperature’s impact on fungal biofilm development has been studied in *C. albicans* [98,99] and *A. fumigatus* [69], little is known about its effects on ECM composition. We employed epifluorescence microscopy to identify ECM molecular constituents. Carbohydrates, nucleic acids, and proteins were observed in the ECM, consistent with findings in other *Aspergillus* biofilms [69,75,76]. Chitin and glycoconjugates, such as those composed of α-D-mannose and α-D-glucose, are major carbohydrate components of the cell wall in *Aspergillus* spp. [84]. The observed staining in cells and extracellular spaces suggests that ECM carbohydrates share the biochemical nature of fungal cell wall components. Mitchel et al. [96] noted that while cell wall and ECM polysaccharides share similarities, they differ in size and branching, having distinct synthesis pathways or undergoing modifications after cleavage from the cell wall. Fluorescence labeling with Flamingo^TM^ confirmed the presence of proteins in the ECM, with the conidia showing more intense fluorescence than the hyphae regardless of the temperature. This likely reflects the hydrophobic protein layer on conidia, which is shed during germination and hyphal development [71,100]. A slight increase in fluorescence was detected in the ECM at 28 °C compared with that at 37 and 42 °C. Additionally, protein inclusions were observed within hyphae at 28 and 37 °C but not at 42 °C. Some proteins identified in *A. fumigatus* ECM are dipeptidylpeptidase V (DPPV), catalase B (CatB), and ribotoxin (ASPF1) [77,101]. Mosier et al. [102] demonstrated that temperature modifies the proteome of biofilms formed by acid mine drainage (AMD) consortia, composed of bacteria, archaea, and filamentous fungi, upregulating proteins involved in amino acid transport and metabolism. Similarly, the development temperature affects the morphology and EPS synthesis in *Clostridium perfringens* biofilms, aiding survival under hostile conditions and host colonization [74,103]. The presence of eDNA in the ECM was detected via propidium iodide staining within the conidia and hyphae, with prominent localization in intercellular spaces at 37 °C. It appeared as diffuse regions colocalizing with glycoconjugates, as described by Shopova et al. [104] in *A. fumigatus*. Although eDNA is a minor ECM component mainly composed of non-coding sequences [105,106], it is crucial for cell adhesion, biofilm cohesion, and structural stability, enhancing resistance to stressors, including antifungal agents [107]. Rajendran et al. [106] showed that eDNA release is phase-dependent and peaks during the maturation phase due to autolysis. In *A. nidulans* biofilm, protease and chitinase activities linked to nutritional stress responses [108] are triggered during biofilm maturation and senescence, degrading ECM polymers and cellular components and supplying alternative carbon and nitrogen sources, as described in bacterial biofilms previously [109,110]. Approximately 14–15% of the ECM in fungal biofilms is composed of lipids [111], with neutral glycerolipids being predominant, followed by sphingolipids [105]. Nile red staining confirmed the presence of lipids in the *A. flavus* biofilm, with minimal fluorescence in the ECM, suggesting that lipids are a minor ECM component, consistent with other fungal models. Higher fluorescence was observed within the conidia, and abundant lipid droplets (LDs) were noted inside the hyphae of the MMe18 isolate across all temperatures. This was further supported by Sudan Black B staining, which revealed that LDs were practically absent in the reference strain ATCC 22546. This difference may be associated with stress adaptation mechanisms or linked to virulence, as previously described in *C. parapsilosis* [112]. Meanwhile, temperature-dependent lipid dynamics have been reported in *Metschnikowia* yeasts, suggesting a connection to stress response mechanisms [113]. Although further research is needed, lipids in the ECM are thought to contribute to structural integrity and adhesion [114]. LDs—amphipathic lipid membranes encasing neutral lipids like triacylglycerols and sterols—are involved in various functions, including stress response, apoptosis, and autophagy [115,116,117,118], and they serve as lipid reservoirs during host invasion as well as in toxin production and secretion [119,120]. Their specific function in biofilm formation, however, remains less understood. Lattif et al. [121] suggested that elevated lipid levels during early biofilm formation enhance surface adhesion, potentially being linked to lipid rafts in the fungal membrane. They also proposed that higher concentrations of polar lipids, such as phospholipids and sphingolipids, may also contribute to antifungal resistance in biofilms. Chang et al. [122] described how the sequestration of lipophilic toxins enhances resistance to these compounds. Our group has documented LDs in *A. terreus* biofilms [93], which is possibly associated with an oleaginous behavior [123]. Oleaginous fungi, known for their lipid production and accumulation, have promising biotechnological applications [113,124,125]. As previously mentioned, filamentous fungal biofilms are characterized by multilayered hyphal networks which, along with ECM production, confer resistance to stressors and adverse conditions [29]. We found that higher temperatures resulted in denser and more compact hyphal networks, as evidenced by the reduced laser penetration during Z-stack imaging at 37 °C and more prominently at 42 °C. This may reflect increased structural integrity at elevated temperatures and could potentially slow antifungal penetration, consistent with reports of molecule sequestration within biofilms [126,127]. Furthermore, we observed that the minimal inhibitory concentration (MIC) of amphotericin B and itraconazole against *A. flavus* showed temperature-dependent variations. Both antifungals were effective against planktonic cells; amphotericin B displayed a twofold higher MIC at 37 °C (4 μg/mL) compared with that at 28 °C and 42 °C (2 μg/mL), while itraconazole was most effective at 42 °C (0.0312 μg/mL) but doubled its effectiveness at 28 °C and 37 °C (0.0625 μg/mL), likely due to temperature-induced stress. Elevated temperatures can impose environmental stress. Stress-induced mutagenesis, leading to antibiotic-resistant phenotypes, has been reported in thermophilic *Bacillus* species [128]. The impact of temperature on the emergence of resistant phenotypes is a topic of significant interest, particularly in the context of climate change, yet it remains largely unexplored. Huang et al. [129] demonstrated that incubation of *Rhodosporidiobolus fluvialis* at 37 °C induces reactive oxygen species (ROS) production, resulting in DNA damage and mutations linked to hypervirulence and pan-resistance. In our study, itraconazole exhibited no antifungal activity against mature biofilms at any tested temperature (inhibitory concentration > 16 μg/mL). Meanwhile, amphotericin B displayed reduced but variable activity, with an inhibitory concentration of 8 μg/mL at 42 °C and 16 μg/mL at 28 °C and 37 °C. This temperature-dependent variability in amphotericin B efficacy is consistent with previous studies, which reported significantly higher MICs for biofilms compared with planktonic cells [33,78,87,130,131,132]. Our group previously reported that *Fusarium solani* biofilms exhibit reduced susceptibility to antifungal drugs and ultraviolet radiation [43]. The mechanisms underlying biofilm-associated resistance have been widely studied. Nett et al. [30] linked increased β-1,3 glucan content in *C. albicans* biofilm cells to enhanced structural integrity and antifungal resistance. Similarly, Rajendran et al. [106] found that eDNA from lysed hyphae in *A. fumigatus* contributes to biofilm resistance, with DNase treatment improving antifungal efficacy. Kowalski et al. [32] demonstrated that oxygen gradients within biofilms enable basal layer cells to survive and resume growth under favorable conditions, conferring resistance to voriconazole and amphotericin B. Similar findings in *C. albicans* highlight the role of persistent cells in antifungal resistance [133].

The next step in our research will focus on identifying the molecular mechanisms underlying conidiation and examining how this process is influenced by the temperature. Is it a response to heat shock stress? Is it just a dispersion mechanism? And which other stressful environmental factors are involved in conidiation? Moreover, we will investigate how temperature affects the regulation of virulence-associated genes in this model, including aflatoxin production.

We aim to characterize the lipid composition within lipid droplets (LDs) and investigate their physiological roles in fungal biofilm formation. Furthermore, we seek to understand the effects of temperature on LD composition and the gene expression involved in their biosynthesis and physiology and explore potential biotechnological applications.

## 5. Conclusions

Our findings confirm that *A. flavus* MMe18 is thermotolerant, with optimal growth at 28 °C and optimal biofilm formation at 37 °C. We suggest that this thermotolerance may be a species-specific trait rather than strain-specific. Moreover, we found that high temperatures enhance conidial germination and conidiation, potentially enhancing fungal dispersion. Conversely, the temperature did not appear to affect the ECM composition of the *A. flavus* biofilms, which primarily consisted of proteins, carbohydrates, and eDNA, highlighting the presence of lipid droplets (LDs) within the hypha of the MMe18 strain. Additionally, biofilm formation significantly increased the MICs of the antifungal agents regardless of the temperature.

## Figures and Tables

**Table 1:** Susceptibility of *Aspergillus flavus* MMe18 to amphotericin B and itraconazole at different incubation temperatures.

	Amphotericin B (μg/mL)		Itraconazole (μg/mL)	
	PK	BF	PK	BF
**28 °C**	2	16	0.0625	>16
**37 °C**	4	16	0.0625	>16
**42 °C**	2	8	0.0312	>16

PK = planktonic cells; BF = mature biofilm.

## Data Availability

Data associated with this study have not been deposited into a publicly available repository and will be made available from the corresponding author on reasonable request.

## Supplementary Materials

The following supporting information can be downloaded at https://www.mdpi.com/article/10.3390/jof11010053/s1. Figure S1: Molecular identification of *A. flavus* MMe18 by Neighbor-Joining phylogenetic tree construction based on the partial β-tubulin gene sequence. Figure S2: Comparison of growth on PDA medium after 4 days of incubation at 28 °C, 37 °C and 42 °C for *A. flavus* ATCC 22546, *A. flavus* MMe18, *A. flavus* PHA, and *A. flavus* MT. Figure S3: *A. flavus* MMe18 conidia germination analysis, adjusting inoculum at (a) 1 × 10^5^ conidia/mL and (b) 1 × 10^7^ conidia/mL. Table S1: Raw data for minimal inhibitory concentration (MIC) determination. Table S2: Raw data for anti-biofilm activity determination by MTT assay.
