# Supplementary material for: The Effect of Temperature over the Growth and Biofilm Formation of the Thermotolerant Aspergillus flavus
Source: J Fungi (Basel). 2025 Jan 10;11(1):53. doi: 10.3390/jof11010053 (PMC11766932; doi:10.3390/jof11010053)
Supplement: Supplementary file 1 [file jof-11-00053-s001.zip › jof-3357453-supplementary.pdf]

Effect of temperature over the growth and biofilm formation of the thermotolerant *Aspergillus flavus*

José Alejandro Hernández-Benítez[1], Brenda Nallely Santos-Ocampo[1], Daniel Genaro Rosas-Ramírez [2], Luis Antonio Bautista-Hernández[3], Víctor Manuel Bautista-de Lucio[3], Néstor Octavio Pérez[4,*], and Aída Verónica Rodríguez-Tovar[1,*]

[1] Departamento de Microbiología, Escuela Nacional de Ciencias Biológicas, Instituto Politécnico Nacional, Prol. Carpio y Plan de Ayala s/n Col. Casco de Santo Tomás, Alcaldía Miguel Hidalgo, C.P. 11340, Ciudad de México, México; jhernandezb2100@alumno.ipn.mx (J.A.H.-B.); bsantoso1400@alumno.ipn.mx (B.N.S.-O.); adramirezg@ipn.mx (A.R.-G.).

[2] Departamento de Química de Biomacromoléculas Instituto de Química, Universidad Nacional Autónoma de México, Av. Universidad 3000, Circuito Exterior s/n, Ciudad Universitaria, Alcaldía Coyoacán, C.P. 04510, Ciudad de México, México; dgrosas@unam.mx

[3] Unidad de Investigación del Instituto de Oftalmología "Fundación de Asistencia Privada Conde de Valenciana I.A.P.", Chimalpopoca 14, Col. Obrera, Alcaldía Cuahutémoc, C.P. 06800, Ciudad de México, México; luis.bautista@institutodeoftalmologia.org (L.A.B.-H); vbautistal@institutodeoftalmologia.org (V.M.B.-L).

[4] Departamento de Investigación y Desarrollo, Probiomed, S.A. de C.V., Cruce de Carreteras Acatzingo-Zumahuacan s/n, Tenancingo, C.P. 52400, Estado de México, México.

* Correspondence: avrodriguez@ipn.mx (A.V.R.-T.), Tel.: +52 5557296000 Ext. 52379; nestor.perez@probiomed.com.mx (N.O.P.).

Table of contents:

Figure S1: Molecular identification of *A. flavus* MMe18 by Neighbor-Joining phylogenetic tree construction based on the partial β-tubulin gene sequence.

Figure S2. Comparison of growth on PDA medium after 4 days of incubation at 28 °C, 37 °C and 42 °C for *A. flavus* ATCC 22546, *A. flavus* MMe18, *A. flavus* PHA, and *A. flavus* MT.

Figure S3. *A. flavus* conidia germination analysis adjusting inoculum at ***a)*** 1x10^{5} conidia/mL and ***b)*** 1x10^{7} conidia/mL.

Table S1: Raw data for minimal inhibitory concentration (MIC) determination.

Table S2: Raw data for anti-biofilm activity determination by MTT assay.

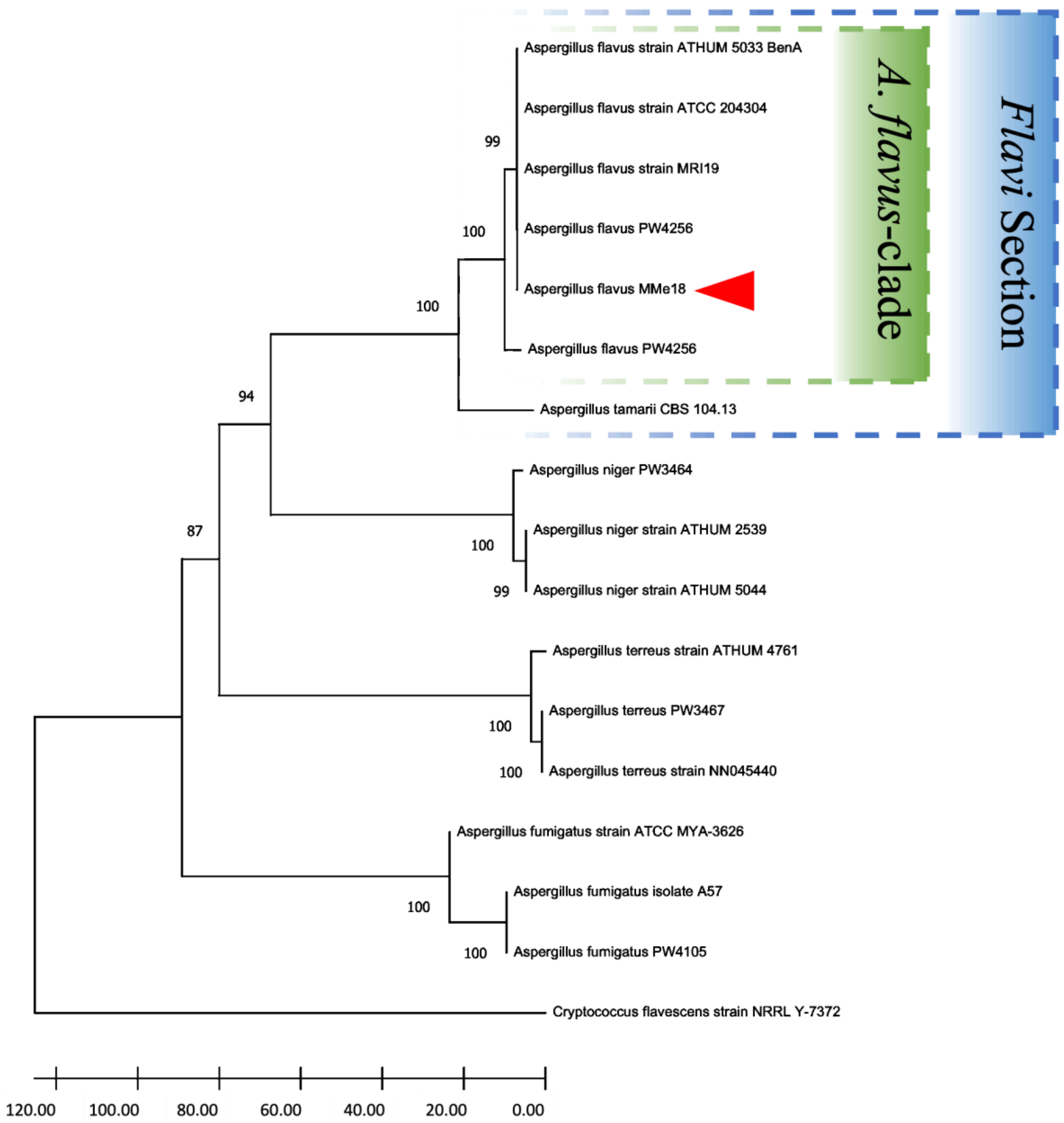

Figure S1. Molecular identification of *A. flavus* MMe18 by Neighbor-Joining phylogenetic tree construction based on the partial β-tubulin gene sequence.

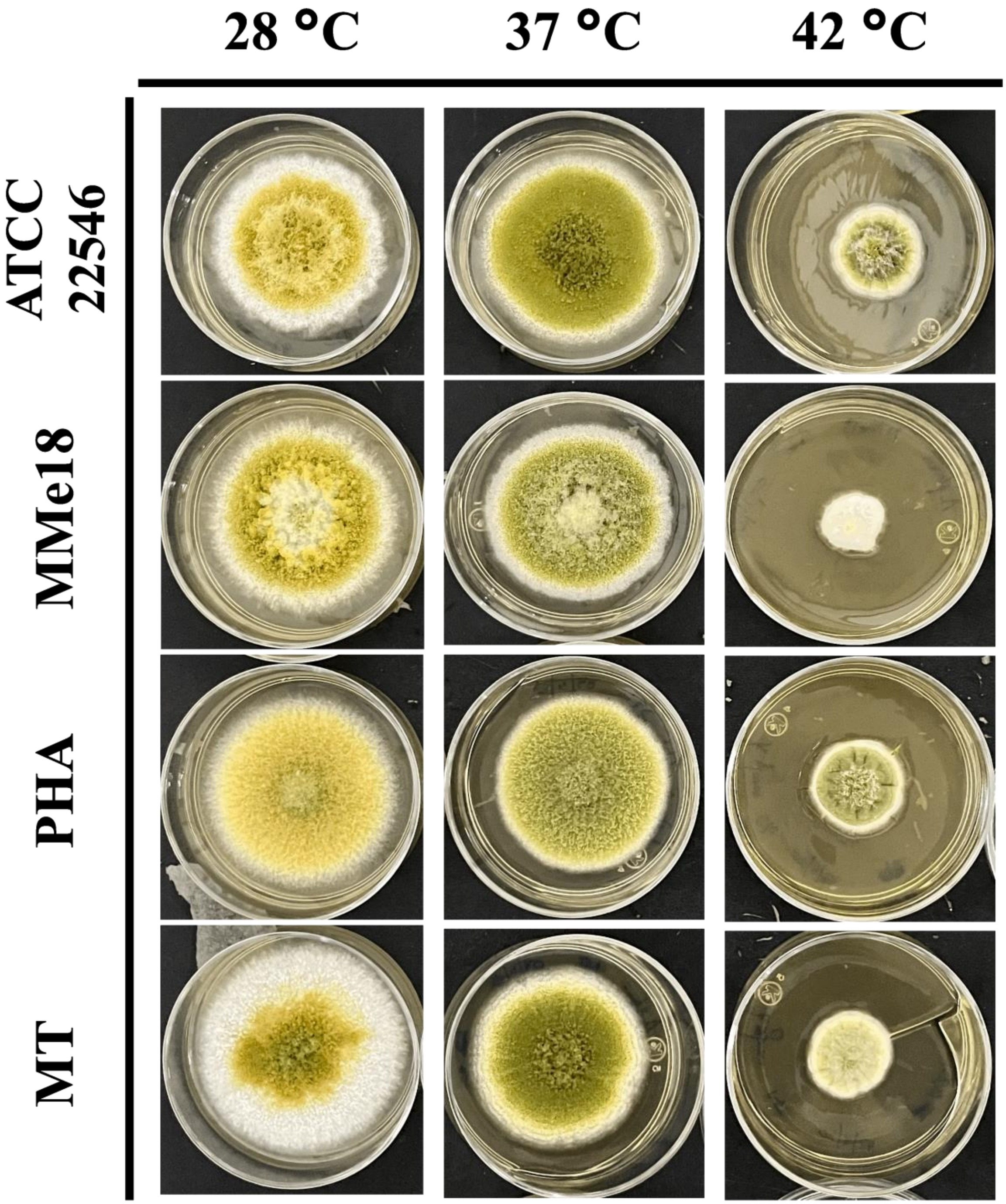

Figure S2. Comparison of growth on PDA medium after 4 days of incubation at 28 °C, 37 °C and 42 °C for *A. flavus* ATCC 22546, *A. flavus* MMe18, *A. flavus* PHA, and *A. flavus* MT.

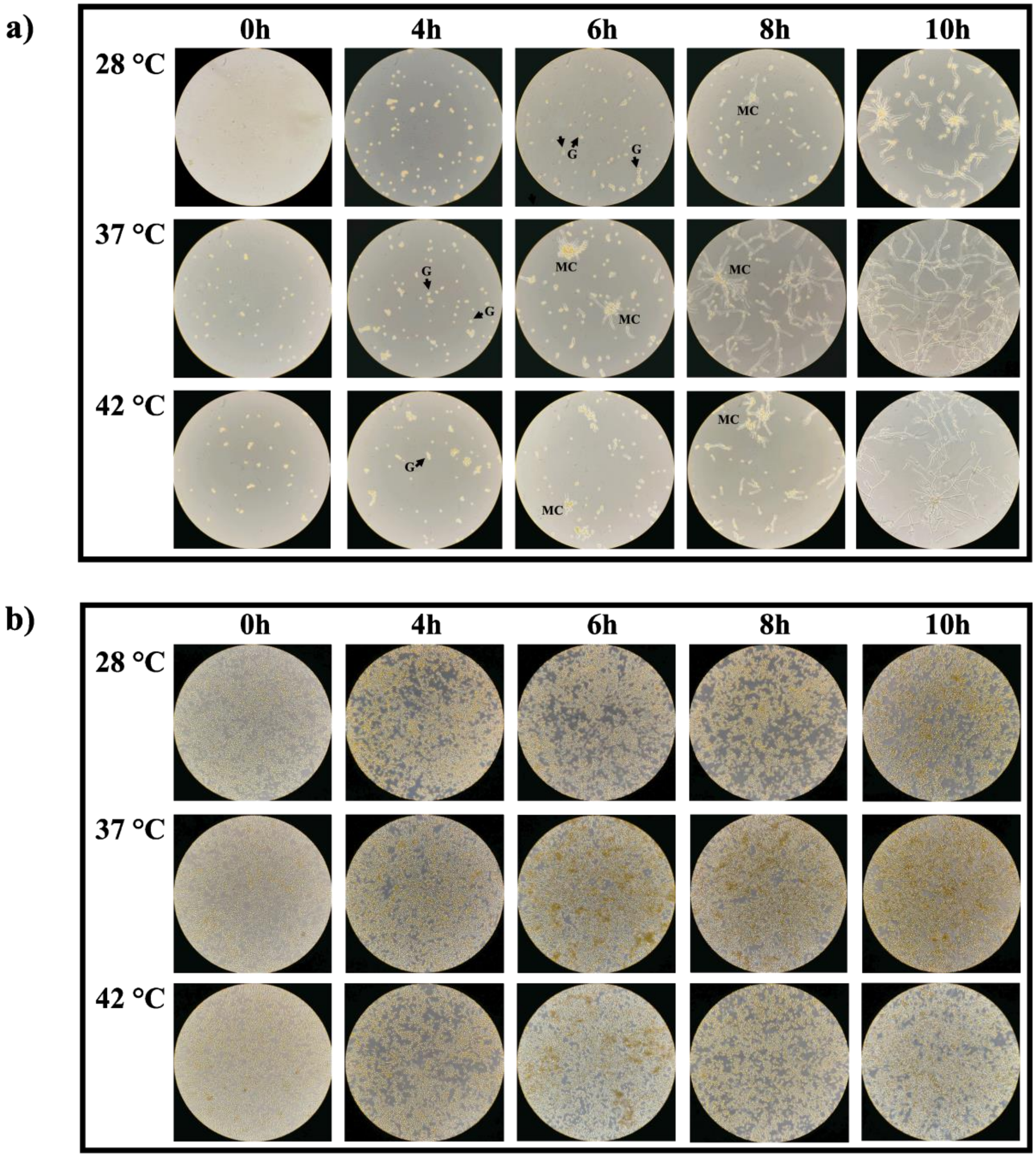

Figure S3. *A. flavus* MMe18 conidia germination analysis, adjusting inoculum at ***a)*** $1x10^5$ conidia/mL and ***b)*** $1x10^7$ conidia/mL.

Table S1: Raw data for minimal inhibitory concentration (MIC) determination.

28 °C	AMPHOTERICIN B (ug/mL)										
CC	0.0312	0.062	0.125	0.25	0.5	1	2	4	8	16	CE
0.329	0.325	0.322	0.338	0.313	0.315	0.245	0.216	0.119	0.031	0.011	0.201
0.328	0.303	0.371	0.360	0.253	0.239	0.216	0.096	0.009	0.050	0.068	0.226
0.311	0.253	0.245	0.268	0.229	0.212	0.237	0.141	0.009	0.011	0.053	0.284
0.291	0.233	0.249	0.316	0.228	0.232	0.235	0.077	0.003	0.009	0.051	0.185
0.311	0.248	0.255	0.264	0.238	0.236	0.216	0.115	0.117	0.034	0.064	0.179
0.303	0.279	0.267	0.267	0.244	0.223	0.192	0.117	-0.023	0.073	0.065	0.188
0.301	0.291	0.322	0.300	0.246	0.248	0.209	0.169	0.123	0.023	0.037	0.198
0.296	0.338	0.338	0.334	0.215	0.225	0.229	0.122	0.105	-0.054	-0.026	0.165
0.419	0.501	0.458	0.398	0.332	0.345	0.290	0.253	0.093	0.121	0.135	0.292
0.411	0.361	0.436	0.347	0.293	0.240	0.230	0.192	0.083	0.129	0.172	0.337
0.439	0.382	0.291	0.308	0.284	0.268	0.264	0.140	0.048	0.086	0.128	0.279
0.414	0.395	0.457	0.324	0.272	0.300	0.265	0.155	0.026	0.040	0.082	0.233
0.455	0.361	0.512	0.329	0.313	0.297	0.274	0.169	0.091	0.102	0.143	0.307
0.445	0.386	0.421	0.319	0.276	0.246	0.254	0.196	0.141	0.092	0.122	0.274
0.523	0.364	0.347	0.313	0.293	0.265	0.235	0.177	0.126	0.082	0.117	0.275
0.494	0.363	0.464	0.372	0.322	0.269	0.276	0.178	0.079	0.105	0.133	0.244

	ITRACONAZOLE (ug/mL)										
CC	0.0312	0.062	0.125	0.25	0.5	1	2	4	8	16	CE
0.447	0.111	0.041	0.025	0.046	0.045	0.049	0.047	0.051	0.047	0.043	0.215
0.434	0.254	0.079	0.086	0.074	0.064	0.061	0.054	0.053	0.105	0.113	0.241
0.383	0.208	0.056	0.048	0.032	0.033	0.020	0.006	0.040	0.073	0.083	0.207
0.307	0.177	0.039	0.032	0.022	0.022	0.008	0.017	0.033	0.067	0.072	0.203
0.368	0.250	0.076	0.075	0.069	0.056	0.046	0.049	0.066	0.103	0.099	0.234
0.416	0.249	0.058	0.065	0.044	0.047	0.038	0.027	0.026	0.080	0.084	0.218
0.412	0.195	0.068	0.062	0.045	0.058	0.029	0.026	0.033	0.083	0.092	0.214
0.316	0.204	0.046	0.044	0.027	0.021	0.015	0.004	0.005	0.060	0.072	0.198
0.437	0.190	-0.013	-0.009	-0.030	-0.025	-0.028	-0.011	-0.044	-0.019	0.084	0.178
0.429	0.234	0.069	0.052	0.050	0.039	0.030	0.008	0.014	0.082	0.071	0.202
0.398	0.186	0.031	0.028	0.018	-0.009	-0.012	-0.015	0.000	0.043	0.034	0.157
0.355	0.162	0.031	0.015	0.010	-0.005	-0.013	-0.011	-0.020	0.044	0.047	0.155
0.597	0.191	0.055	0.059	0.038	0.023	0.006	0.000	0.002	0.051	0.042	0.181
0.540	0.173	0.048	0.032	0.029	0.016	-0.007	-0.002	-0.024	0.039	0.025	0.188
0.493	0.209	0.052	0.042	0.027	0.018	0.002	-0.011	-0.008	0.041	0.035	0.183
0.528	0.198	0.048	0.042	0.018	0.023	0.004	-0.007	-0.023	0.038	0.018	0.178

37 °C	AMPHOTERICIN B (ug/mL)										
CC	0.0312	0.062	0.125	0.25	0.5	1	2	4	8	16	CE

0.304	0.268	0.273	0.369	-0.051	0.355	0.257	0.272	0.046	0.059	0.061	0.190
0.327	0.350	0.350	0.358	0.329	0.337	0.310	0.279	-0.007	0.053	0.070	0.228
0.321	0.294	0.325	0.318	0.272	0.273	0.293	0.285	-0.021	0.026	0.043	0.193
0.301	0.326	0.271	0.263	0.253	0.283	0.255	0.167	-0.007	0.030	0.051	0.181
0.312	0.294	0.311	0.319	0.289	0.273	0.258	0.314	0.019	0.052	0.065	0.204
0.320	0.304	0.317	0.321	0.301	0.292	0.286	0.298	-0.022	0.030	0.036	0.212
0.314	0.300	0.313	0.301	0.286	0.300	0.277	0.200	-0.018	0.025	0.056	0.209
0.280	0.291	0.293	0.295	0.296	0.295	0.278	0.261	0.176	0.009	0.006	0.194
0.315	0.357	0.373	0.379	0.374	0.398	0.339	0.276	0.143	0.108	0.126	0.265
0.415	0.397	0.357	0.387	0.351	0.334	0.361	0.340	0.167	0.127	0.155	0.302
0.379	0.303	0.310	0.323	0.292	0.303	0.313	0.305	0.149	0.109	0.125	0.264
0.437	0.327	0.310	0.335	0.286	0.271	0.284	0.438	0.023	0.058	0.080	0.215
0.432	0.370	0.337	0.348	0.320	0.326	0.341	0.298	0.100	0.128	0.144	0.279
0.465	0.332	0.363	0.351	0.323	0.343	0.387	0.338	0.101	0.097	0.147	0.254
0.422	0.342	0.335	0.303	0.346	0.341	0.331	0.304	0.068	0.100	0.115	0.250
0.408	0.344	0.370	0.371	0.355	0.368	0.355	0.276	0.073	0.100	0.116	0.234

	ITRACONAZOLE (ug/mL)										
**CC**	**0.0312**	**0.062**	**0.125**	**0.25**	**0.5**	**1**	**2**	**4**	**8**	**16**	**CE**
0.305	0.286	-0.006	0.104	0.079	-0.001	0.003	0.011	-0.024	0.102	0.082	0.271
0.364	0.266	0.079	0.102	0.076	0.075	0.073	0.068	0.054	0.096	0.078	0.307
0.331	0.237	0.047	0.053	0.043	0.046	0.042	0.041	0.036	0.072	0.051	0.269
0.287	0.198	0.006	0.006	-0.012	-0.009	-0.007	-0.019	-0.014	0.026	0.018	0.215
0.337	0.238	0.071	0.078	0.063	0.061	0.067	0.057	0.046	0.089	0.084	0.289
0.335	0.234	0.049	0.057	0.044	0.046	0.047	0.049	0.022	0.073	0.053	0.268
0.345	0.208	0.059	0.061	0.043	0.046	0.050	0.042	0.045	0.061	0.045	0.264
0.281	0.187	0.032	0.042	0.026	0.028	0.033	0.021	0.017	0.051	0.026	0.246
0.305	0.227	-0.070	-0.059	-0.072	-0.073	-0.072	-0.062	-0.070	0.019	0.020	0.198
0.322	0.206	0.013	0.011	0.006	-0.013	-0.013	-0.011	-0.021	0.026	0.024	0.213
0.300	0.219	-0.013	-0.016	-0.025	-0.049	-0.044	-0.049	-0.025	-0.001	0.016	0.192
0.309	0.158	-0.005	-0.007	-0.019	-0.029	-0.027	-0.037	-0.021	0.015	0.033	0.222
0.392	0.216	0.001	0.003	-0.012	-0.024	-0.019	-0.037	-0.026	0.005	0.020	0.200
0.363	0.165	-0.004	-0.006	-0.014	-0.024	-0.020	-0.027	-0.029	0.011	0.036	0.199
0.317	0.152	-0.011	0.004	-0.013	-0.027	-0.029	-0.024	-0.032	0.004	0.019	0.198
0.291	0.159	0.001	-0.006	-0.023	-0.029	-0.030	-0.040	-0.043	0.007	0.023	0.197

**42 °C**	AMPHOTERICIN B (ug/mL)										
**CC**	**0.0312**	**0.062**	**0.125**	**0.25**	**0.5**	**1**	**2**	**4**	**8**	**16**	**CE**
0.343	0.288	0.247	0.297	0.258	0.251	0.190	0.081	0.053	0.066	0.090	0.257
0.366	0.375	0.345	0.349	0.314	0.297	0.217	0.066	0.060	0.085	0.117	0.277
0.331	0.319	0.281	0.272	0.201	0.253	0.144	0.060	0.040	0.057	0.083	0.239
0.370	0.273	0.207	0.273	0.231	0.233	0.141	0.043	0.019	0.024	0.042	0.191

0.310	0.315	0.287	0.264	0.277	0.228	0.168	0.107	0.070	0.076	0.094	0.248
0.469	0.297	0.268	0.256	0.270	0.238	0.146	0.077	0.031	0.051	0.094	0.236
0.249	0.268	0.267	0.276	0.249	0.230	0.161	0.162	0.040	0.045	0.068	0.236
0.307	0.335	0.240	0.349	0.237	0.232	0.136	0.136	0.039	0.044	0.067	0.241
0.208	0.382	0.355	0.285	0.276	0.280	0.185	0.096	0.036	-0.005	0.303	0.233
0.238	0.318	0.301	0.308	0.315	0.300	0.255	0.147	0.027	0.102	0.114	0.251
0.296	0.417	0.232	0.326	0.269	0.282	0.196	0.044	0.002	0.068	0.082	0.235
0.264	0.323	0.252	0.252	0.195	0.199	0.159	0.018	-0.020	0.033	0.039	0.208
0.282	0.454	0.421	0.294	0.225	0.224	0.174	0.074	0.021	0.093	0.095	0.248
0.281	0.460	0.372	0.283	0.210	0.231	0.176	0.043	-0.002	0.075	0.077	0.211
0.280	0.494	0.321	0.361	0.223	0.220	0.167	0.082	0.005	0.076	0.068	0.213
0.181	0.332	0.266	0.231	0.181	0.206	0.149	0.117	-0.023	0.035	0.050	0.185

	ITRACONAZOLE (ug/mL)										
**CC**	**0.0312**	**0.062**	**0.125**	**0.25**	**0.5**	**1**	**2**	**4**	**8**	**16**	**CE**
0.303	0.100	0.051	0.060	0.063	0.057	0.061	0.043	-0.079	0.038	0.002	0.293
0.293	0.093	0.084	0.093	0.077	0.081	0.084	0.082	-0.016	0.076	0.027	0.282
0.292	0.065	0.044	0.058	0.048	0.054	0.047	0.052	-0.084	0.037	0.002	0.262
0.335	0.020	0.006	0.008	0.004	-0.013	-0.002	-0.015	-0.099	0.000	-0.009	0.219
0.417	0.083	0.073	0.084	0.061	0.065	0.055	0.050	-0.066	0.061	0.008	0.280
0.297	0.068	0.054	0.067	0.052	0.050	0.042	0.040	-0.071	0.042	-0.015	0.255
0.365	0.082	0.058	0.062	0.046	0.043	0.053	0.045	-0.008	0.035	-0.001	0.251
0.346	0.046	0.030	0.039	0.028	0.034	0.037	0.036	-0.010	0.017	-0.007	0.227
0.369	0.057	-0.010	-0.018	-0.015	-0.017	-0.008	-0.013	-0.008	0.024	-0.005	0.229
0.405	0.048	0.032	0.030	0.019	0.022	0.026	0.025	0.000	0.046	0.047	0.253
0.265	0.002	-0.006	-0.015	-0.021	-0.028	-0.041	-0.016	-0.042	-0.009	-0.012	0.109
0.277	0.006	-0.010	-0.017	-0.021	-0.030	-0.039	-0.039	-0.067	-0.034	-0.021	0.181
0.362	0.029	0.018	0.017	0.013	0.009	-0.007	0.000	-0.039	0.016	0.006	0.224
0.407	0.020	0.010	0.008	-0.006	-0.007	-0.009	-0.008	-0.062	0.000	0.005	0.215
0.297	0.023	0.011	0.008	-0.008	-0.004	-0.005	0.000	-0.045	0.009	0.007	0.220
0.263	0.022	-0.007	-0.005	-0.027	-0.024	-0.017	-0.018	-0.060	-0.001	0.000	0.196

Table S2: Raw data for anti-biofilm activity determination by MTT assay.

28 °C		AMPHOTERICIN B (ug/mL)										
0h	CC	0.0312	0.062	0.125	0.25	0.5	1	2	4	8	16	CE
	0.893	0.262	0.238	0.388	0.221	0.272	0.082	0.018	0.023	0.049	0.068	0.331
	0.798	0.489	0.225	0.149	0.265	0.249	0.045	0.009	0.022	0.010	0.037	0.307
	0.717	0.341	0.269	0.250	0.246	0.285	0.078	0.014	0.001	0.031	0.033	0.256
	0.746	0.297	0.257	0.277	0.314	0.269	0.160	0.012	-0.003	0.009	0.056	0.232
	0.934	0.451	0.309	0.162	0.118	0.063	0.073	0.079	0.095	0.106	0.257	0.256
	0.577	0.324	0.177	0.084	0.068	0.076	0.037	0.065	0.061	0.087	0.148	0.233
	0.829	0.189	0.123	0.069	0.034	0.017	0.028	0.021	0.015	0.055	0.053	0.242
	0.765	0.147	0.109	0.071	0.011	0.009	0.015	0.007	0.001	0.027	0.068	0.226
		ITRACONAZOLE (ug/mL)										

28 °C		AMPHOTERICIN B (ug/mL)										
12h	CC	0.0312	0.062	0.125	0.25	0.5	1	2	4	8	16	CE
	1.101	0.498	0.453	0.449	0.486	0.345	0.526	0.831	0.250	0.164	0.218	0.209
	1.035	0.436	0.510	0.444	0.591	0.373	0.669	0.473	0.253	0.159	0.207	0.236
	1.058	0.484	0.479	0.539	0.680	0.591	0.500	0.288	0.221	0.155	0.167	0.195
	0.991	0.391	0.312	0.553	0.402	0.554	0.548	0.427	0.472	0.170	0.195	0.188
	1.116	0.486	0.497	0.490	0.516	0.498	0.481	0.522	0.515	0.526	0.435	0.215
	1.092	0.673	0.480	0.250	0.544	0.458	0.466	0.196	0.506	0.435	0.407	0.207
	1.117	0.551	0.488	0.738	0.604	0.060	0.423	0.593	0.563	0.465	0.569	0.204
	1.149	0.515	0.526	0.763	0.674	0.582	0.727	0.644	0.700	0.613	0.531	0.188
		ITRACONAZOLE (ug/mL)										

28 °C		AMPHOTERICIN B (ug/mL)										
24h	CC	0.0312	0.062	0.125	0.25	0.5	1	2	4	8	16	CE
	0.841	0.631	0.668	0.359	0.414	0.452	0.403	0.359	0.356	0.249	0.249	0.169
	0.991	0.746	0.568	0.416	0.366	0.366	0.429	0.392	0.376	0.374	0.201	0.187
	0.829	0.643	0.669	0.526	0.423	0.262	0.889	0.375	0.361	0.336	0.175	0.175
	0.708	0.613	0.644	0.645	0.444	0.521	0.449	0.392	0.224	0.489	0.243	0.217
	0.835	0.949	0.799	0.431	0.768	0.712	0.610	0.549	0.325	0.422	0.444	0.184
	0.945	0.647	0.711	0.663	0.796	0.441	0.656	0.654	0.486	0.585	0.630	0.177
	0.830	0.738	0.888	0.791	0.802	0.778	0.426	0.532	0.614	0.483	0.403	0.180
	0.787	0.918	0.721	0.629	0.629	0.775	0.524	0.581	0.853	1.005	0.613	0.186
		ITRACONAZOLE (ug/mL)										

37 °C		AMPHOTERICIN B (ug/mL)										
0h	CC	0.0312	0.062	0.125	0.25	0.5	1	2	4	8	16	CE

0.662	0.407	0.364	0.325	0.315	0.323	0.448	0.387	0.115	0.065	0.075	0.272
0.978	0.452	0.441	0.317	0.347	0.341	0.484	0.471	0.066	0.074	0.083	0.316
0.917	0.451	0.568	0.286	0.260	0.312	0.359	0.463	0.011	0.037	0.065	0.224
0.898	0.388	0.342	0.266	0.294	0.338	0.326	0.445	-0.009	0.043	0.040	0.197
1.016	0.481	0.367	0.250	0.124	0.089	0.079	0.069	0.051	0.056	0.091	0.239
0.874	0.419	0.291	0.366	0.495	0.064	0.077	0.040	0.091	0.121	0.047	0.214
0.869	0.538	0.309	0.315	0.108	0.072	0.057	0.034	0.027	0.041	0.038	0.262
0.578	0.460	0.304	0.331	0.115	0.068	0.023	0.013	0.016	0.080	0.208	0.241
	**ITRACONAZOLE (ug/mL)**										

**37 °C**		**AMPHOTERICIN B (ug/mL)**										
**12h**	**CC**	**0.0312**	**0.062**	**0.125**	**0.25**	**0.5**	**1**	**2**	**4**	**8**	**16**	**CE**
	0.713	0.424	0.516	0.463	0.456	0.303	0.592	0.714	0.745	0.601	0.521	0.242
	0.974	0.466	0.449	0.466	0.424	0.404	0.894	0.381	0.671	0.397	0.450	0.255
	0.939	0.498	0.640	0.442	0.728	0.352	0.644	0.688	0.531	0.501	0.403	0.196
	0.859	0.439	0.413	0.482	0.381	0.291	0.766	0.470	0.552	0.434	0.306	0.187
	1.015	0.504	0.566	0.451	0.363	0.148	0.131	0.187	0.106	0.182	0.119	0.210
	0.843	0.629	0.609	0.570	0.481	0.174	0.092	0.101	0.152	0.171	0.104	0.204
	0.867	0.703	0.703	0.555	0.366	0.192	0.213	0.131	0.169	0.189	0.245	0.201
	0.604	0.516	0.567	0.541	0.412	0.323	0.130	0.101	0.271	0.184	0.092	0.203
		**ITRACONAZOLE (ug/mL)**										

**37 °C**		**AMPHOTERICIN B (ug/mL)**										
**24h**	**CC**	**0.0312**	**0.062**	**0.125**	**0.25**	**0.5**	**1**	**2**	**4**	**8**	**16**	**CE**
	0.765	0.4	0.426	0.395	0.366	0.44	0.373	0.527	0.246	0.466	0.474	0.194
	0.913	0.292	0.375	0.341	0.405	0.475	0.342	0.537	0.256	0.357	0.32	0.235
	0.769	0.26	0.472	0.334	0.357	0.273	0.297	0.418	0.13	0.315	0.09	0.184
	0.792	0.234	0.398	0.352	0.363	0.364	0.271	0.468	0.167	0.315	0.088	0.228
	1.040	0.427	0.64	0.476	0.747	0.564	0.361	0.537	0.36	0.715	0.413	0.213
	0.952	0.378	0.561	0.474	0.462	0.656	0.392	0.578	0.517	0.398	0.389	0.217
	0.860	0.418	0.59	0.447	0.634	0.463	0.398	0.474	0.472	0.457	0.432	0.233
	0.653	0.373	0.49	0.489	0.428	0.577	0.439	0.44	0.374	0.439	0.405	0.208
		**ITRACONAZOLE (ug/mL)**										

**42 °C**		**AMPHOTERICIN B (ug/mL)**										
**0h**	**CC**	**0.0312**	**0.062**	**0.125**	**0.25**	**0.5**	**1**	**2**	**4**	**8**	**16**	**CE**
	0.746	0.272	0.290	0.551	0.346	0.310	0.133	0.093	-0.020	-0.001	0.050	0.187
	0.584	0.285	0.324	0.269	0.309	0.257	0.135	0.044	-0.029	0.023	0.040	0.260
	0.521	0.259	0.416	0.270	0.308	0.253	0.150	0.029	-0.031	0.002	0.018	0.214

0.667	0.276	0.241	0.329	0.319	0.238	0.102	0.028	-0.012	0.022	0.030	0.230
0.555	0.413	0.180	0.034	0.039	0.025	0.015	0.062	0.000	0.015	0.009	0.228
0.506	0.377	0.189	0.023	0.033	0.014	0.012	-0.010	-0.034	-0.013	-0.001	0.213
0.526	0.356	0.170	0.042	0.010	-0.006	-0.003	-0.002	-0.045	-0.005	-0.013	0.250
0.578	0.352	0.306	0.023	0.024	0.001	-0.015	-0.039	-0.058	-0.029	0.002	0.221
	**ITRACONAZOLE (ug/mL)**										

**42 °C**		**AMPHOTERICIN B (ug/mL)**										
**12h**	**CC**	**0.0312**	**0.062**	**0.125**	**0.25**	**0.5**	**1**	**2**	**4**	**8**	**16**	**CE**
	0.514	0.261	0.350	0.264	0.445	0.382	0.344	0.344	0.375	0.061	0.010	0.220
	0.508	0.461	0.333	0.369	0.318	0.365	0.320	0.428	0.142	0.077	0.020	0.247
	0.527	0.428	0.343	0.306	0.355	0.327	0.296	0.387	-0.010	0.036	0.021	0.197
	0.489	0.447	0.379	0.254	0.237	0.279	0.348	0.251	0.053	0.022	-0.007	0.223
	0.654	0.349	0.558	0.338	0.215	0.137	0.090	0.150	0.062	0.144	0.067	0.259
	0.520	0.357	0.339	0.282	0.184	0.030	0.051	0.039	-0.002	0.056	0.035	0.236
	0.491	0.415	0.348	0.300	0.274	0.022	0.025	0.026	0.008	0.014	0.020	0.257
	0.467	0.295	0.428	0.332	0.368	0.055	0.025	-0.009	-0.004	0.030	0.017	0.243
		**ITRACONAZOLE (ug/mL)**										

**42 °C**		**AMPHOTERICIN B (ug/mL)**										
**24h**	**CC**	**0.0312**	**0.062**	**0.125**	**0.25**	**0.5**	**1**	**2**	**4**	**8**	**16**	**CE**
	0.482	0.462	0.340	0.306	0.393	0.329	0.354	0.330	0.206	0.120	0.138	0.209
	0.611	0.531	0.384	0.338	0.340	0.315	0.379	0.333	0.225	0.104	0.120	0.286
	0.554	0.601	0.497	0.406	0.361	0.654	0.298	0.498	0.320	0.168	0.090	0.215
	0.496	0.288	0.328	0.306	0.323	0.379	0.249	0.211	0.167	0.018	0.223	0.242
	0.637	0.648	0.445	0.545	0.358	0.515	0.374	0.383	0.326	0.504	0.498	0.270
	0.577	0.569	0.329	0.577	0.541	0.320	0.502	0.353	0.308	0.380	0.347	0.252
	0.546	0.397	0.307	0.418	0.307	0.299	0.287	0.303	0.323	0.343	0.310	0.261
	0.583	0.269	0.284	0.290	0.337	0.263	0.282	0.309	0.228	0.290	0.307	0.230
		**ITRACONAZOLE (ug/mL)**										
